# Coupling of co-transcriptional splicing and 3’ end Pol II pausing during termination in Arabidopsis

**DOI:** 10.1186/s13059-023-03050-4

**Published:** 2023-09-11

**Authors:** Sixian Zhou, Fengli Zhao, Danling Zhu, Qiqi Zhang, Ziwei Dai, Zhe Wu

**Affiliations:** 1 Harbin Institute of Technology, Harbin, 150001 China; 2 Key Laboratory of Molecular Design for Plant Cell Factory of Guangdong Higher Education Institutes, Institute of Plant and Food Science, Department of Biology, School of Life Sciences, Southern University of Science and Technology, Shenzhen, 518055 China; 3 Department of Biology, School of Life Sciences, Southern University of Science and Technology, Shenzhen, 518055 China

**Keywords:** Pol II transcription, Transcription termination, Co-transcriptional splicing, Exon numbers, Plants

## Abstract

**Background:**

In Arabidopsis, RNA Polymerase II (Pol II) often pauses within a few hundred base pairs downstream of the polyadenylation site, reflecting efficient transcriptional termination, but how such pausing is regulated remains largely elusive.

**Result:**

Here, we analyze Pol II dynamics at 3’ ends by combining comprehensive experiments with mathematical modelling. We generate high-resolution serine 2 phosphorylated (Ser2P) Pol II positioning data specifically enriched at 3’ ends and define a 3’ end pause index (3’PI). The position but not the extent of the 3’ end pause correlates with the termination window size. The 3’PI is not decreased but even mildly increased in the termination deficient mutant xrn3, indicating 3’ end pause is a regulatory step early during the termination and before XRN3-mediated RNA decay that releases Pol II. Unexpectedly, 3’PI is closely associated with gene exon numbers and co-transcriptional splicing efficiency. Multiple exons genes often display stronger 3’ end pauses and more efficient on-chromatin splicing than genes with fewer exons. Chemical inhibition of splicing strongly reduces the 3’PI and disrupts its correlation with exon numbers but does not globally impact 3’ end readthrough levels. These results are further confirmed by fitting Pol II positioning data with a mathematical model, which enables the estimation of parameters that define Pol II dynamics.

**Conclusion:**

Our work highlights that the number of exons via co-transcriptional splicing is a major determinant of Pol II pausing levels at the 3’ end of genes in plants.

**Supplementary Information:**

The online version contains supplementary material available at 10.1186/s13059-023-03050-4.

## Background

Control of gene expression at the transcriptional level is of vital importance. Transcription can be divided into three stages, initiation, elongation, and termination; among these, termination is the least understood [1, 2]. Accumulating evidence in plants indicates that the regulation of termination and/or pre-mRNA polyadenylation could play important roles in plant development and plant-environment interactions [3–6]. Albeit its importance, the mechanism underlying Pol II regulation at gene 3’ end is poorly understood in plants. Notably, flowering plants such as Arabidopsis harbor putative RNA 3’ end processing factors that are plant-specific, potentiating the presence of plant-specific mechanism of Pol II regulation at 3’ end.

For decades, the termination mechanism of Pol II in mammalian cells has been summarized by two alternative models, the torpedo model [7, 8] and the allosteric model [9]. In the torpedo model, cleavage occurs after Pol II passes through the polyadenylation signal. A 5’ to 3’ exonuclease then degrades the nascent RNA associated with transcribing Pol II and dissociates Pol II from the DNA template [7, 8]. In the allosteric model, Pol II elongation through the polyadenylation signal leads to a conformational change of Pol II or the dissociation of antitermination factors from Pol II, thus promoting termination [9]. Both models are supported by substantial evidence, while the latest evidence suggests a combined model (or the sitting duck model) is evident [10, 11]. In such a model, polyadenylation signal dependent-cleavage leads to dephosphorylation of SPT5 by PNUTS/PP1 and causes an allosteric switch of Pol II. Pol II slows down, allowing the XRN2 torpedo to catch up with transcribing Pol II and releasing it from the DNA template [10, 11].

To what extent the above mechanism holds in plants is largely unknown. In Arabidopsis, mutation of XRN3, a plant homolog of human XRN2, leads to increased readthrough transcripts [12–14] and an accumulation of RNAs with their 5’ end aligned at the cleavage site, supporting the torpedo model [15]. However, whether XRN3 affects Pol II pausing at 3’ end is unclear. Other known regulators of termination and or RNA 3’ end processing in plants include FCA [16, 17], FPA [18, 19], FY [20, 21], Cstf64, Cstf77 [22, 23], HLP1 [24], CPSF30 [3, 25], and BORDER [26]; intriguingly, most are also regulators of plant flowering time [5]. To date, the biochemical function, as well as the integration mechanism among these different proteins in plant Pol II termination remains elusive.

In mammals, high precision mapping of Pol II position was enabled by mammalian native elongation transcript sequencing (mNET-seq) [27]. In mNET-seq, Pol II is immunoprecipitated together with its associated 3’region of the nascent RNA by using monoclonal antibody that recognize the YSPTSPS repeats of endogenous Pol II CTD with or without phosphorylation at a certain cite. Pol II position in single-nucleotide resolution is then defined by mapping the 3’end of the immunoprecipitated RNA. Notably, due to the conservation of YSPTSPS repeats of Pol II CTD among eukaryotes [28], the antibody used in mNET-seq could be applicable for a broad range of species. Indeed, by applying mNET-seq in Arabidopsis (named as plant NET-seq, pNET-seq) [29], it was showed that the Pol II with unphosphorylated CTD is mainly enriched at gene 5’end, while Pol II with Ser5 phosphorylated (Ser5P) CTD is closely related with the spliceosome as splicing intermediates that are presumably protected by spliceosome are detected in Ser5P Pol II pNET-seq [29] and Total Pol II NET-seq data (known as plaNET-seq) [30]. Notably, Ser2P Pol II is mainly associated with active elongation and transcription termination [27, 31–33]. Arabidopsis Pol II, especially the Ser2P form, displays a sharp peak post polyadenylation site often within a few hundred base pairs [29, 30, 34], indicating plant Pol II pauses effectively at gene 3’end. In addition, the position of Pol II peak at gene 3’end coincide with the end of the termination window as measured by long-read sequencing [15], indicating Pol II 3’end pause is associated with termination. Consistent with the above notion, a plant homolog of Negative Elongation Factor [35] called BORDER [26] was reported to promote both 3’end Pol II pause (as detected in Ser2P, Ser5P and Total Pol II ChIP-seq) and termination in Arabidopsis.

Evidence across species, tissues, and cell types showed that transcription is tightly coupled with RNA processing, such that events like exon splicing occur simultaneously with Pol II elongation [36–43]. In Arabidopsis, introns are mainly removed co-transcriptionally [36, 37]. In addition, it was previously reported that co-transcriptional splicing efficiency correlates with the numbers of introns at genes, indicating widespread presence of cooperativity among the splicing of different exons within individual genes [36, 37]. Hence, the mechanistic basis of such cooperativity is unclear. Consistently, neighboring introns of mammalian genes also tend to be removed concurrently [43, 44]. Of note, in yeast and mammalian cells, splicing is also coupled with Pol II dynamics at gene 3’ end. 3’ end pause of Pol II is mainly observed in yeast genes with efficient splicing [45, 46]. Early evidence based on individual genes suggests that recruitment of splicing factors and correct assembly of the spliceosome are coupled to transcription termination in mammalian cells [47]. Consistently, the latest long-read PacBio sequencing data of nascent RNA showed inefficient co-transcriptional splicing is associated with defective 3’ end cleavage in mammals [43]. Whether and how splicing is coupled with Pol II at gene 3’ end in plants is unknown.

## Results

### Ser2P Pol II pauses at 3’ end of a subset of genes in Arabidopsis

**Fig. 1:**
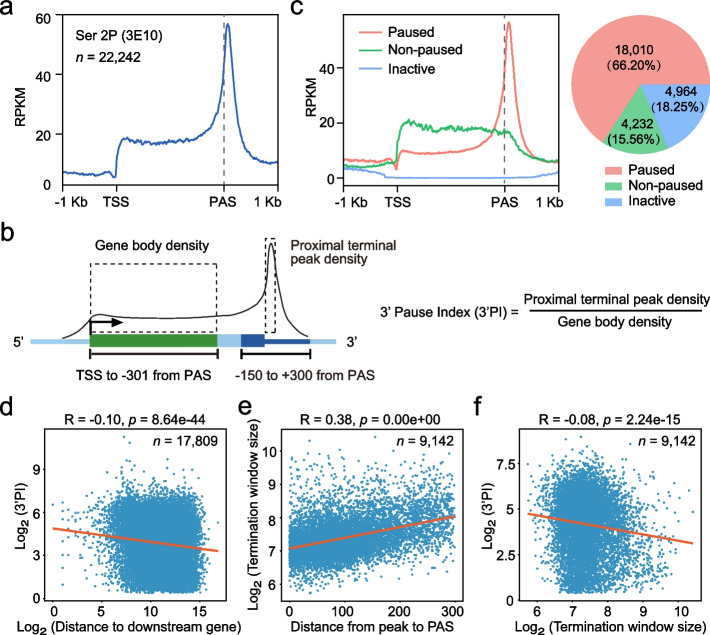
Quantification of Pol II pausing at gene 3’ end. **a** Metagene plot showing Pol II profile across protein-coding genes based on Ser2P Pol II pNET-seq data obtained with 3E10 antibody. Only the expressed protein-coding genes (TPM ≥ 1 based on the Ser2P Pol II pNET-seq data) are used for analysis. TSS, transcription start site. PAS, polyadenylation site. **b** Diagram showing the definition of the 3’ end pause index (3’PI). **c** Metagene profile of Ser2P Pol II at paused, non-paused, and inactive genes. Paused and non-paused genes were defined by 3’PI, and inactive genes were determined based on pNET-seq data (TPM < 1 at the gene region). **d** Scatter plot indicating the relationship between 3’PI and the distance to downstream gene. Only 3’ paused genes that does not overlap with other genes are used for this analysis. **e** Scatter plot showing the relationship between termination window size and the distance from 3’ end peak to PAS. **f** Scatter plot showing the relationship between 3’PI and the termination window size. For **e** and **f**, only genes with known termination window size and also 3’PI are used for the analysis. For **d** to **f**, the Spearman correlation coefficient is shown above the plot. The orange line indicates the trendline fitted based on the least squares method

To investigate the Pol II dynamics at gene 3’ end, we performed pNET-seq with an antibody (3E10) that specifically targets Pol II with a Ser2P CTD (Fig. 1a, Additional file 1: Fig. S1a), as we previously tested at the Arabidopsis *FLC* gene [33]. 3E10 antibody is raised against the YSphPTSPS peptide, the sequence of which is highly conserved in Pol II CTD of both plants and mammals [28]. As we expected, the Ser2P pNET-seq data by 3E10 antibody showed solo enrichment at gene 3’ end (Fig. 1a) with no pronounced peak at gene 5’ end. Such a pattern is consistent with what we observed at *FLC* gene [33] and also the Ser2P Pol II ChIP-seq profile as generated by using another antibody [26]. Of note, previously published Ser2P pNET-seq data by using CMA602 antibody also showed certain enrichment at gene 5’end, in addition to the enrichment at gene 3’ end (Additional file 1: Fig. S1b, c) [29]. We reasoned that such difference is likely due to different antibody specificity towards Ser2P Pol II CTD (see “Discussion”). Nevertheless, given its tight association with gene 3’ end, we used our Ser2P Pol II pNET-seq data for further analysis. Both the previous data [29] and our data suggest Pol II pausing at gene 3’ end is profoundly associated with Pol II with Ser2P CTD, a feature that is conserved in plants and animals.

Next, we wondered to quantify the relative extent of 3’ end Pol II pause at different genes. Of note, the levels of Pol II at any defined position of a gene is determined by Pol II initiation rate over its elongation rate at that position [33, 48]. Thus, the net peak height of the 3’ end Pol II peak is not a good estimate of the 3’ end pause levels and is strongly influenced by differential Pol II initiation rate at different genes [33, 48]. We therefore quantify the level of Pol II pausing by defining a 3’ end Pausing Index (3’PI) (Fig. 1b). We first defined the gene body (from transcription start site (TSS) to −301 of polyadenylation site (PAS)) and 3’ end region (− 150 to + 300 bp of the PAS) (For the choice of the 3’end region, see “Methods”). Using 50-bp sliding windows, we further identified a peak window within the 3’ end region for each expressed gene. Genes whose pNET-seq read density at the 3’ end peak window is significantly higher than the gene body are considered as 3’ end paused genes, and a 3’PI is calculated as the ratio between two values (Fig. 1b). The 3’PI reflects the extent of the 3’ end pause and is independent of Pol II initiation rate. Notably, among the expressed genes, there are 18,010 or 4232 genes with or without a 3’ end pause, respectively, suggesting 3’ end pause can be detected at the majority but not all of the genes (Fig. 1c; Additional file 1: Fig. S1d, e). In addition, we found that 3’PI only weakly related to the length of intergenic regions (*R* =  −0.1), suggesting the latter is not a major factor in determining Pol II pausing level at 3’end (Fig. 1d; Additional file 1: Fig. S2a).

### The position while not the extent of 3’ end pause correlates with termination window size

We wondered how 3’ end pause is related to transcriptional termination. By long-reads sequencing, the termination window size (TWS) of Pol II transcription in Arabidopsis was defined previously [15], a value describes how far Pol II travels downstream of poly (A) site before it is released from chromatin. We, therefore, investigated the relationship between 3’ end pause and TWS. Consistent with previous observations [15], the position of 3’ end pause is well-matched with TWS such that the bigger the TWS, the longer distance between 3’ end pause and poly (A) site can be observed (Fig. 1e). Thus, the 3’ end pause is indeed tightly associated with termination. Comparatively, we found that the 3’PI does not or only very weakly correlate with TWS at the genome-wide scale (Fig. 1f), as judged by a correlation coefficient of −0.08. Consistently, genes with similar TWS can be very different for their levels of 3’ end pause (Additional file 1: Fig. S2b). Thus, the position while not the extent of 3’ end pause correlates with Pol II TWS, suggesting there might be other layers in regulating the levels of 3’ end pause, in addition to transcription termination.

### 3’ end pause is not due to XRN3-mediated RNA decay

**Fig. 2:**
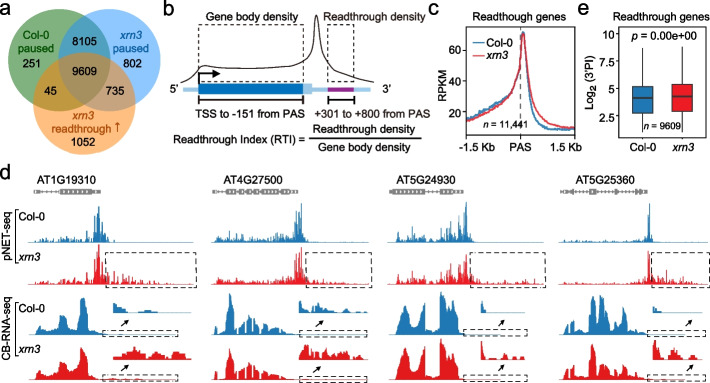
Pol II pause at gene 3’end is not mediated by XRN3. **a** Venn diagram showing the overlap among paused genes in Col-0, paused genes in *xrn3*, and genes that display increased readthrough in *xrn3* compared to Col-0. **b** Diagram showing the definition of readthrough index (RTI). **c** Metagene plot showing Pol II profiles at 3’ end of genes with increased readthrough in *xrn3* compared to Col-0. **d** Typical examples from the gene group as shown in **c**. Genome browser tracks showing the normalized counts from pNET-seq data (1 nt resolution) and chromatin-bound RNA-seq data. The gray dashed box highlights the region with readthrough. The black arrow indicates the zoom-in view of the region with readthrough. **e** Box plot demonstrates the distribution of 3’PI in Col-0 and *xrn3* at genes with increased readthrough in *xrn3* compared to Col-0. The line in the box plot indicates the median, box edges represent the first and third quartiles, and the whiskers extend to the farthest data points within 1.5 × interquartile range outside box edges. *p* value was calculated based on a paired Wilcoxon test

The above data suggests 3’ end pause is a feature innately associated with termination, but its extent is not a simple reflection of termination efficiency as measured by TWS. Thus, we further investigated 3’ end Pol II dynamics upon loss of XRN3, an exonuclease in Arabidopsis that promote Pol II termination by degrade cleaved transcripts during termination [12–15]. Ser2P Pol II pNET-seq data was obtained for *xrn3*. In both Col-0 and *xrn3*, genes with 3’ end pause are largely overlapped (Fig. 2a). Next, we identified genes that display increased levels of transcription readthrough in *xrn3*. We first quantified the level of readthrough in both genotypes using the readthrough index (RTI) (Fig. 2b). As defined previously [27], RTI represents the level of readthrough transcript and is independent of Pol II transcription level (initiation rate). We then identified genes that display significantly different levels of RTI between Col-0 and *xrn3*. This analysis revealed 11,441 genes with increased readthrough in *xrn3*; among them, 84% (9609/11441) display 3’ end pause in both Col-0 and *xrn3* (Fig. 2a), suggesting loss of XRN3 leads to defective termination at the global level (Additional file 1: Fig. S3a), consistent with previous observations [12–15]. Surprisingly, at the 11,441 genes, metagene analysis showed that neither the position nor the level of 3’ end Pol II peak is affected in *xrn3* (Fig. 2c, Additional file 1: Fig. S3b, c, d). Consistently, such a pattern can be observed at many individual genes (Fig. 2d). Notably, the 3’PIs are even slightly increased in *xrn3* compared with Col-0, either at all the protein-coding genes (Additional file 1: Fig. S3e) or at 9609 genes that display both 3’ end Pol II pause and increased readthrough in *xrn3* (Fig. 2e), suggesting XRN3 may aid the releasing of Pol II from 3’ end pause (see “Discussion”). Taken together, XRN3-mediated decay of cleaved transcript is not the cause for Pol II pausing at gene 3’ end in Arabidopsis.

### 3’ end Pol II pause correlates with the number of exons

**Fig. 3:**
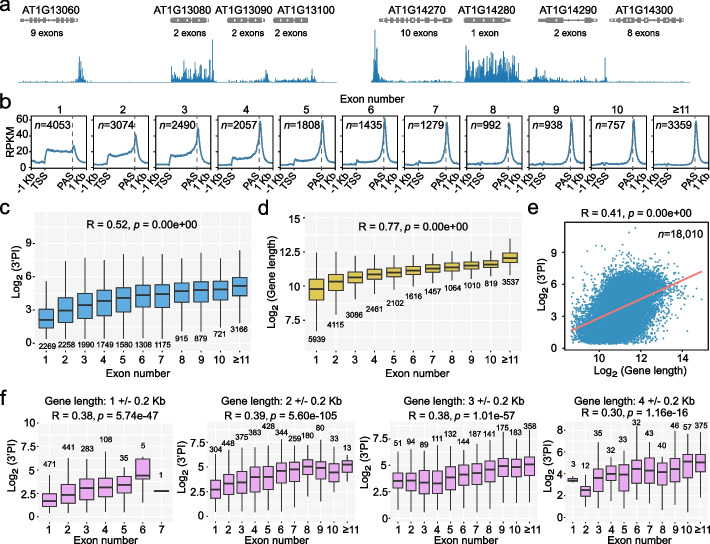
Pol II pause at gene 3’ end correlates with the number of exons. **a** Pol II profile of gene examples with different exon numbers. **b** Metagene plots demonstrate Pol II profiles at gene groups with fixed exon numbers. Only the expressed protein-coding genes (TPM ≥ 1 based on the Ser2P Pol II pNET-seq data) are used for this analysis. **c** Box plots demonstrate the relationship between 3’PI and exon numbers. **d** Box plots demonstrate the relationship between gene length and exon numbers. All protein-coding genes in TAIR10 are used for this analysis. **e** Scatter plot demonstrates the correlation between 3’PI and gene length. The orange line indicated the trendline fitted based on the least squares method. **f** Box plots demonstrate the relationship between 3’PI and exon numbers in different gene groups with fixed gene length. For **c** to **f**, the Spearman correlation coefficient is indicated above the plot. R value and significance tests were performed by “stats.spearmanr” function from the “scipy” package in Python. For box plots, the line indicates the median, box edges represent the first and third quartiles, and the whiskers extend to the farthest data points within 1.5 × interquartile range outside box edges

The fact that genes with similar TWS could have very different levels of 3’PI drives us to explore if 3’ end pause could have other means apart from termination. Visual inspection of Ser2P pNET-seq data revealed an interesting trend that 3’ end Pol II peak is frequently observed at genes with multiple exons; in contrast, 3’ end Pol II peak is often missing at single-exon genes or genes with only two or three exons (Fig. 3a). Indeed, 3’ end paused genes have substantially more exons than non-paused genes (Additional file 1: Fig. S4a, b). Among 18,010 paused genes in Col-0 (Fig. 1), there are 2269 (12.6%) single-exon genes, while among 4232 non-paused genes; there are 1957 (46.2%) single-exon genes. In addition, the Pol II level at the gene body showed an opposite trend comparing with 3’ end, such that Pol II signal is captured more frequently at the body of genes with only small numbers of exons (Fig. 3a, b). As we expected, 3’PI correlates strongly with the number of exons; the higher the number of exons, the stronger the 3’ end pause (Fig. 3c). Notably, such correlation can also be observed using previously published Pol II Ser2P data (Additional file 1: Fig. S4c). In addition, mutation of XRN3 does not disrupt such correlation (Additional file 1: Fig. S4d).

Next, given that the numbers of exons correlate with gene length (Fig. 3d), we did further analysis to distinguish which one of the two factors is more related to 3’PI. 3’PI is correlated with the numbers of exons even when the gene length is fixed (Fig. 3e, f). In comparison, 3’PI is not correlated with gene length at genes with a fixed number of exons when the exon numbers are above three (Additional file 1: Fig. S5a). Of note, gene length correlates with 3’PI most significantly for single-exon genes. Similar results were obtained when considering the relationship between 3’PI and exon length at gene groups with fixed numbers of exons (Additional file 1: Fig. S5b). Therefore, between gene length and the numbers of exons, the latter has a significantly stronger contribution in determining the correlation with 3’PI for multiple exon genes.

### Efficient co-transcriptional splicing and 3’ end pause mutually promote their correlations with exon numbers

**Fig. 4:**
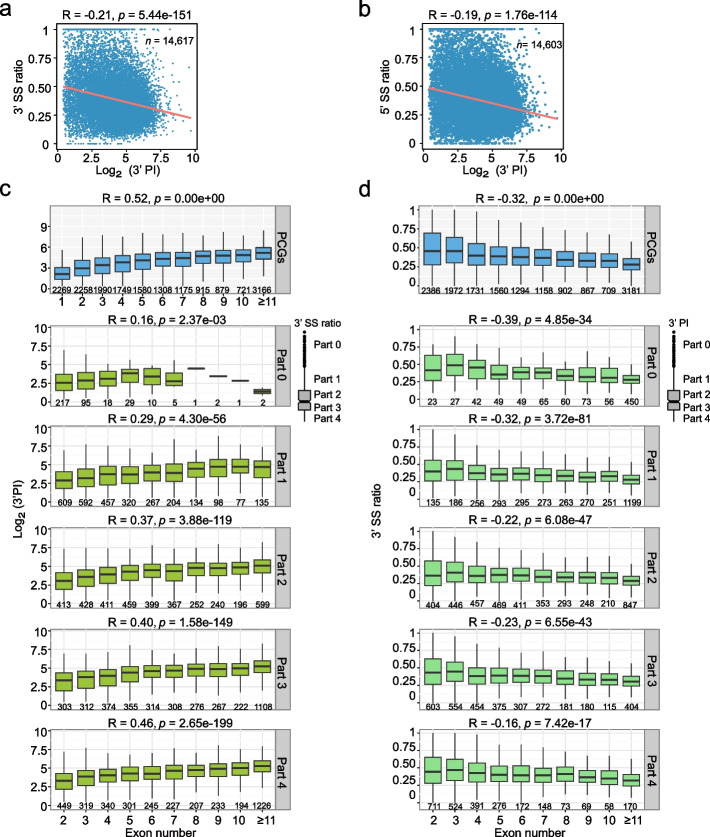
Splicing efficiency and 3’ end Pol II pause mutually promote their correlations with exon numbers. **a**, **b** Scatter plot indicating the correlation between 3’PI and 3’SS ratio (left) or 5’SS ratio (right). Only the genes with both SS ratio and 3’PI are included. The Spearman correlation coefficient was shown above the plot. The orange line indicates the trendline fitted based on the least squares method. **c** Box plots demonstrate the relationship between 3’PI and exon numbers in gene groups with different levels of 3’SS ratio. The plot in blue demonstrates the situation of all protein-coding genes. Protein-coding genes were divided into five groups according to average 3’SS ratios of genes (part 0 to part 4, from high to low), and the relationship between 3’PI and exon numbers was plotted separately for each group. **d** Box plots demonstrate the relationship between the 3’SS ratio and exon numbers in gene groups with different levels of 3’PI. The plot in blue demonstrates the situation of all protein-coding genes. Protein-coding genes were divided into five groups according to the 3’PI (part 0 to part 4, from high to low), and the relationship between the 3’SS ratio and exon numbers was plotted separately for each group. For **c** and **d**, The Spearman correlation coefficient is indicated above each plot. Gene numbers of each group were shown below the boxes

We previously found that the average efficiency of co-transcriptional splicing (CTS) correlates with the number of introns at genes in Arabidopsis [37]. Thus, we investigated the relationship between 3’PI and CTS. We performed chromatin-bound RNA-seq and defined the CTS efficiency in Col-0 by using 5’ and 3’ splice site ratio (5’SS and 3’SS ratio), a value reflecting intron inclusion level on chromatin (Additional file 1: Fig. S6a) [37]. As we expected, 3’PI also correlates with the average CTS efficiency at the global level (Fig. 4a, b). Given that 3’PI, exon numbers and CTS are correlated among each other, we further explored the contribution of CTS towards the correlation between 3’PI and exon numbers. We separate genes into five groups according to their average 5’SS or 3’SS ratios and then investigate the relationship between 3’PI and exon numbers. Notably, compared with the situation in whole genome, the correlation between 3’PI and exon numbers becomes weaker in gene groups with less efficient co-transcriptional splicing (Fig. 4c; Additional file 1: Fig. S6b). Generally, the more efficient CTS (low 5’SS or 3’SS ratios), the stronger the correlation between 3’PI and exon numbers can be observed. We also investigated how CTS efficiency and exon numbers are related in gene groups with fixed levels of 3’PI. Intriguingly, 3’PI also positively impact the correlation between CTS efficiency and exon numbers, such that the higher levels of 3’PI, the better negative correlation between 5’SS (or 3’SS ratios) and exon numbers can be observed (Fig. 4d; Additional file 1: Fig. S6c). Taken together, 3’ end pause, CTS and exon numbers are tightly connected; 3’ end pause and efficient CTS mutually promote their correlations with exon numbers.

### Inhibition of spliceosome assembly by herboxidiene treatment reduces 3’ end pausing and disrupts the correlation between exon numbers and 3’ end pause

**Fig. 5:**
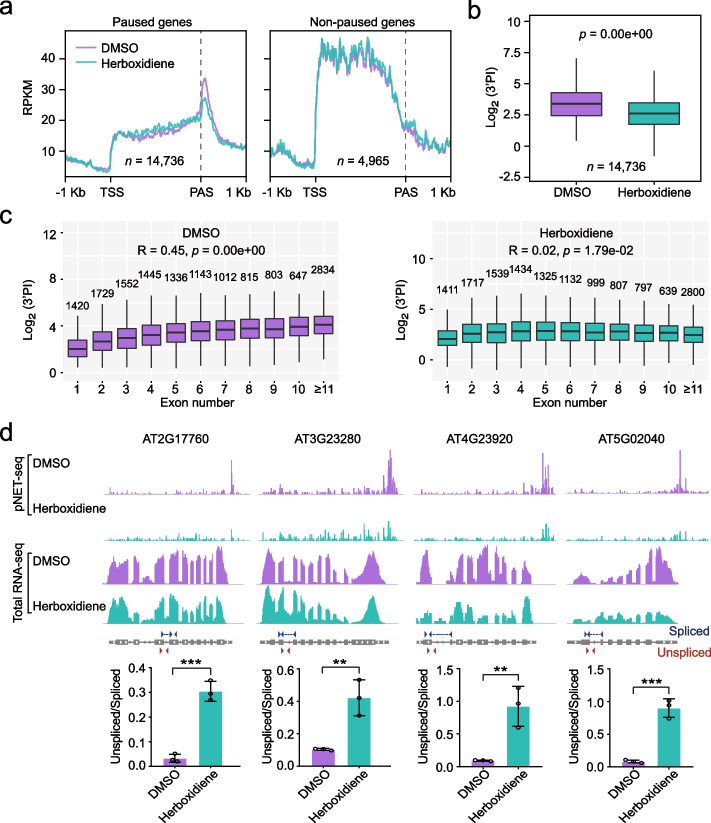
Inhibition of splicing reduces the extent of 3’ end Pol II pause. **a** Metagene plots showing Pol II profiles of paused and non-paused genes in samples treated with DMSO (the control) or herboxidiene (splicing inhibitor). Only the genes that are active (TPM ≥ 1 based on the Ser2P Pol II pNET-seq data) in both DMSO and herboxidiene-treated samples are used for this analysis. Paused and non-paused genes were defined based on the 3’PI of the control sample treated with DMSO. **b** Box plot comparison of the 3’PI between DMSO-treated and herboxidiene-treated samples. 3’PI of genes that display 3’ end pause both in DMSO- and herboxidiene-treated samples are shown. *p* value was calculated based on a paired Wilcoxon test. **c** The relationship between 3’PI and exon numbers in samples treated with DMSO (left) or herboxidiene (right). The Spearman correlation coefficient is indicated at the top of the plot. Gene numbers of each group are indicated above the boxes. **d** Gene examples of which the herboxidiene treatment reduces the 3’ end pause. Genome browser tracks indicate the normalized counts from pNET-seq data and total RNA-seq data. Bar charts below the figure demonstrate the qPCR validation of altered splicing efficiency. Primer locations are labelled on the top (spliced primer) or the bottom (unspliced primer) of the gene structure. Data are presented as mean ± SD (*n* = 3). Asterisk indicates a significant difference based on a two-tailed *t*-test (* p < 0.05, ** p < 0.01, *** p < 0.001)

The above data suggests that efficient CTS positively impacts 3’ end pause, while 3’ end pause also contributes to the cooperativity of splicing at multiple exon genes. Next, we tested if there is any causal relationship between splicing and 3’ end pause. We treated the Arabidopsis seedlings with herboxidiene, a chemical that blocks the catalytic spliceosome formation post the recruitment of U2 snRNP to the pre-mRNA [49, 50]. Compared with control (DMSO treated), after 1-h treatment with herboxidiene, splicing is globally inhibited as measured by the 5’SS or 3’SS ratios in total RNA-seq (Additional file 1: Fig. S7a, b). Among 9659 genes of which the expression can be detected in total RNA-seq dataset (TPM > 1), 8373 genes showed significant splicing inhibition after herboxidiene treatment compared with the control, as judged by gene average intron inclusion levels (Additional file 1: Fig. S7c). The rest 1286 genes remained unaltered for their gene splicing. We then performed Ser2P Pol II pNET-seq under the same condition. There are 14,736 genes display 3’ end pause in DMSO control group and pNET-seq signals in both DMSO and herboxidiene-treated samples (Fig. 5a, b). Astonishingly, at these genes, both the Pol II peak at 3’ end and the 3’PI are dramatically reduced after herboxidiene treatment (Fig. 5a, b; Additional file 1: Fig. S7d). As a control, herboxidiene treatment did not affect Pol II profiles on genes without 3’ end Pol II pause as identified in DMSO-treated control (Fig. 5a). Thus, herboxidiene does not affect transcription initiation and elongation generally in plants, consistent with previous reports [49–51]. Furthermore, the correlation between 3’PI and exon numbers is diminished after the herboxidiene treatment (Fig. 5c), suggesting 3’PI correlate with exon numbers largely due to splicing. Consistently, similar effects can be observed at many individual genes (Fig. 5d). In addition, after the herboxidiene treatment, the readthrough levels are not changed at the majority of genes (7262 genes, Additional file 1: Fig. S8). At the same time, 1398 and 359 genes showed downregulated and upregulated levels of readthrough after the treatment, respectively (Additional file 1: Fig. S8). Thus, splicing inhibition has limited effect on Pol II readthrough, especially compared with its impact on 3’ end pause. Taken together, the above data suggest that 3’ end pause is functionally connected with splicing, such that catalytic spliceosome assembly is required for 3’ end pause (Fig. 5), while the 3’ end pause, in turn, likely promotes splicing at multiple exons genes (Fig. 4).

### Simulation of Pol II pNET-seq data by mathematical modelling confirms that 3’ end pausing time is a function of exon numbers

**Fig. 6:**
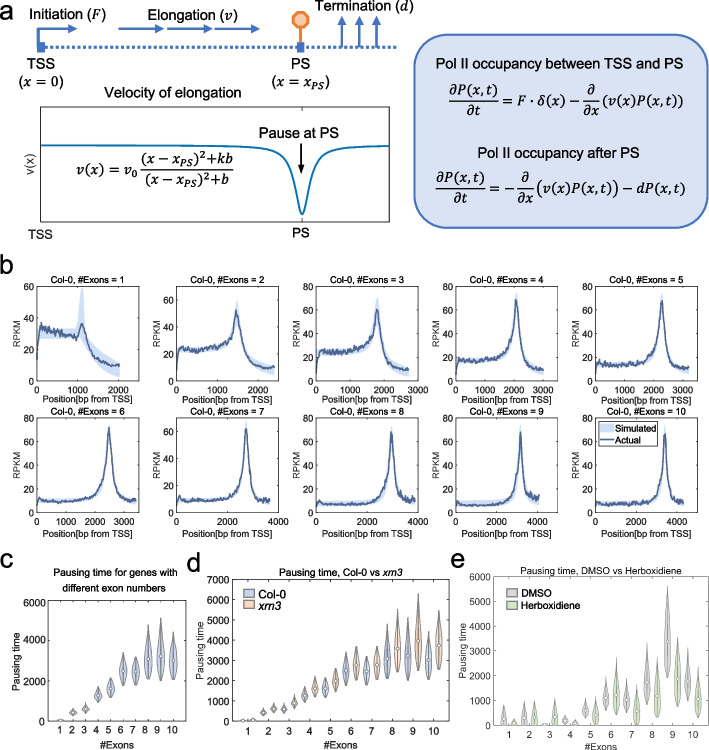
Mathematical model of Pol II pausing dynamics. **a** Scheme of the mathematical model. The velocity of elongation ($v$) is assumed to be a function of the distance to the TSS ($x$), and the pause is modelled by a decrease of velocity at the pausing site (PS) near the PAS. Pol II occupancy between TSS and PS and that after the PS is then computed by solving two partial differential equations that link the Pol II occupancy to the velocity of elongation. **b** Comparison of model-predicted and actual Pol II occupancy profiles in Col-0. Curves indicate the average Pol II occupancy profiles for all genes with a specific exon number estimated from the pNET-seq data, and the shaded areas indicate model-predicted ranges of the Pol II occupancy based on parameters estimated by fitting the experimental data. **c** Comparison of model-predicted 3’ end pausing time across genes with different numbers of exons in Col-0. The pausing time was computed by the decrease in the total time for the entire process of transcription initiation, elongation, and termination to complete if there was no 3’ end pause. **d** Comparison of model-predicted 3’ end pausing time between Col-0 and the *xrn3* mutant for genes with different numbers of exons. **e** Comparison of model-predicted 3’ end pausing time between the DMSO- and herboxidiene-treated groups for genes with different numbers of exons

To investigate the dynamics of the 3’ end pause in different groups of genes and quantitatively characterize the time of 3’ end pause in different genes and conditions, we developed a partial differential equation (PDE)-based mathematical model that links the Pol II occupancy profiles obtained from the pNET-seq data to the initiation, elongation, and termination of transcription (Fig. 6a, “Methods”). The model hypothesizes that the velocity of transcript elongation decreases near the pausing site (PS) that locates close to the PAS, and the extent of the decrease of velocity and the range of genomic region affected by it, as well as the rates of transcription initiation and termination, change with the number of exons included in the genes. Pol II occupancy was then theoretically predicted by solving the steady state of the model. We estimated model parameters by fitting the model to the average Pol II occupancy profiles in genes with different numbers of exons and found that the model is highly consistent with the experimental data (Fig. 6b, Additional file 1: Fig. S9). It is worth noting that the location of the pausing site is only determined by fitting the model to pNET-seq data. Thus, Pol II elongation dynamics that are not reflected in the pNET-seq data, are not accessed or reflected in our simulation (e.g., Pol II peak before PAS as captured in the GRO-seq data [29, 34]). We define a 3’ end pausing time by computing the increase in total time required for completion of transcription in the scenario with 3’ end pause compared to the scenario without 3’ end pause (see “Methods”) and calculated the pausing time for genes with different exon numbers. Consistent with our previous results, the pausing time is significantly higher in genes with more exons (one-way ANOVA *p* value < 10^−323^, Fig. 6c). We next estimated model parameters and 3’ end pausing time using pNET-seq data in the *xrn3* mutant and compared its pausing time with that in Col-0. We found that the 3’ end pausing time in the *xrn3* also increases with exon numbers (Fig. 6d). Consistent with results based on 3’PI (Fig. 2e), 3’ end pausing time is slightly longer in *xrn3* than that in Col-0 (Fig. 6d). Thus, XRN3 may play a positive role in releasing paused Pol II (see “Discussion”). We also compared the model-predicted 3’ end pausing time between the herboxidiene and DMSO-treated groups and found that treatment with herboxidiene significantly reduced the 3’ end pausing time (Fig. 6e, one-sided Wilcoxon’s rank-sum *p* value < 10^−323^ for genes with 1, 4, 5, or more than six exons). Interestingly, comparison of the model parameters across different conditions and genes with different numbers of exons suggests that the rates of initiation and termination also change with the number of exons (Additional file 1: Fig. S10, see “Discussion”). Taken together, the mathematical model enables quantification of 3’ end pausing time based on the pNET-seq data and further supports the relationship between 3’ end pause of Pol II and exon numbers by means of splicing.

## Discussion

In this study, we investigated the Pol II dynamics and its regulation at gene 3’ end in Arabidopsis. We found the Pol II with Ser2P CTD mainly enriched at gene 3’ end (Fig. 1a, b), consistent with the results obtained in yeast and mammalian cells. Interestingly, such a pattern is more clearly observed in our pNET-seq data (3E10 antibody) than in the previous published Ser2P pNET-seq data (CMA602 antibody, no longer commercially available), in which substantial enrichment at gene 5’ end can also be observed (Additional file 1: Fig. S1b). Considering the conservation of Pol II in eukaryotes, we reasoned that such a difference between our data and previous data is likely due to different antibodies. Indeed, it was showed previously that CMA602 antibody may cross react with Pol II CTD that displays both Ser5P and Ser7P [52]. In addition, CMA602 is raised against SYSPTSPSYSphosPTSPSYSP peptide [52], the sequence of which has less copies within Arabidopsis Pol II CTD compared with the YSphosPTSPS that used to raise 3E10 [28]. Overall, our results suggest 3E10 antibody is ideal for profiling Pol II at gene 3’ end in plants.

The Pol II peak at gene 3’ end can be observed for both Ser2P CTD Pol II and Ser5P CTD Pol II (Fig. 1a; Additional file 1: Fig. S1b, c), suggesting 3’ end pause of Pol II is a general feature of Pol II at gene 3’ end. Meanwhile, the highly specific enrichment of Ser2P CTD Pol II at gene 3’ end in our data enabled us to look at the features and regulation of 3’ end pause in detail. We found the position of 3’ end pause is tightly associated with TWS, suggesting 3’ end pause is a feature innately linked with transcription termination (Fig. 1e). Most intriguingly, mutation of XRN3 does not affect the position of Pol II pause but causes a slight increase of pausing time, as showed by 3’PI data (Fig. 2e; Additional file 1: Fig. S3e) and also mathematical modelling (Fig. 6d). Thus, our data suggest Pol II pause likely occurs prior to XRN3 engagement, and XRN3 may aid the release of paused Pol II at 3’ end. Interestingly, XRN3 could also impact elongation as the Pol II level in the gene body region slightly decreases globally in *xrn3-3* (Additional file 1: Figs. S3a, S12). The exact reason for this remains unknown, but such a phenomenon reflects the coupling between elongation and termination. It is also worth noting that a relatively weak allele *xrn3-3* is used in our study, thus we cannot exclude the possibility that a stronger effect on 3’ pause can be observed in a stronger allele of XRN3 such as *xrn3-8* [12]. Nevertheless, our data suggest that termination control in plants involves both 3’ end Pol II pause and exonuclease-mediated RNA decay, a scenario similar to the combined model of termination (or the Sitting Duck model) recently proposed in mammalian cells. In this scenario, Pol II change its conformation and pauses at gene 3’ end; Pol II behaves similar as a “sitting duck” when it pauses, waiting for the XRN3 torpedo to release it from the DNA template. The exact structure and implication of Pol II conformation at gene 3’ end remains a challenge to define. Pol II conformation at 3’ end could be much more complex than just the Ser2P of Pol II CTD, although Ser2P is highly enriched at 3’ end.

Our work revealed tight coupling between 3’ end Pol II pause and exon splicing at multiple exons genes. It was shown previously that 3’ end Pol II pause mainly occurs at genes with multiple exons in yeast [45], a unicellular organism in which genes mostly has only a single exon. Our data showed this feature was kept and further evolved in multicellular organisms like Arabidopsis (Fig. 3a–c), the genome of which is dominated by multiple exons genes. Indeed, the 3’ end pause contributes to the mutually promoting nature of splicing among different exons within individual genes (Fig. 4d, Additional file 1: Fig. S6c), a feature highlighted in plants. The 3’ end pause likely gives substantial time for efficient on-chromatin splicing, which is especially critical for plant genes given their unique structures. Compared with mammalian genes, plant genes and yeast genes both have generally much shorter intron, while plant genome harbors lots more multiple exon genes than yeast. Thus, assuming similar Pol II elongation rate and spliceosome assembly kinetics among species, it is particularly challenging for effective co-transcriptional or on-chromatin splicing in plants. Based on our data, the 3’ end pause of Pol II provides an explanation for this. Consistent with the data at individual genes in mammals [47], we found that 3’ end pause largely requires effective spliceosome assembly in plants at a genome-wide scale (Fig. 5). Thus, a considerable proportion of on-chromatin splicing could be finished when Pol II pauses at gene 3’ end. Indeed, consistently, it was also reported previously that splicing inhibition did not change the local Pol II dynamics at the splice junction of individual genes [51]. It is currently unknown if there are particular factors to signal splicing status to Pol II at gene 3' end or that the spliceosome is simply in touch with Pol II and holding it at 3’ end. These possibilities remain to be distinguished in the future.

We showed that mathematical modelling is highly effective in resolving Pol II dynamics when combined with high-resolution Pol II occupancy data such as pNET-seq (Fig. 6). Indeed, for most multicellular organisms, studying Pol II dynamics in real time is extremely challenging and often remains unfeasible. Our modelling results confirmed that 3’PI is a good estimate of Pol II pausing extent (Fig. 6), while modelling enables a more accurate estimate of Pol II pausing time. For example, the mutation of human exonuclease (XRN2) leads to pileup of Pol II at 3’ end [10, 11], a phenomenon that is not observed in Arabidopsis; hence, our modelling results showed that mutation of exonuclease causes an increase of Pol II pausing time at 3’ end (Fig. 6d). Our modelling results showed the 3’ end pausing time is most strongly affected by the number of exons, compared with *xrn3*, suggesting exon numbers by means of splicing is a major determinant of 3’ end pause (Fig. 6c–e). It is worth noting that to fit the data, Pol II dynamics at the gene body region also need to be altered as a function of exon numbers (Additional file 1: Fig. S10). Indeed, at genes with more exons, the gene body density of Pol II is also lower (Fig. 6b), suggesting either the Pol II initiation rate or the elongation rate, or both parameters need to be tuned when the exon number increases. In our current model, given that we fixed the Pol II elongation rate at the gene body, it is necessary to change Pol II initiation rate to fit the gene body Pol II profiles at gene groups with different numbers of exons (Additional file 1: Fig. S10). Thus, it would be interesting to test in the future if transcriptional initiation and elongation rate are generally coordinated between each other and are functions of exon numbers, a scenario that is similar to what we observed at Arabidopsis *FLC* previously [33]. Our modelling results also indicate that Pol II’s release rate from the template when it travels post the pausing site (d, Additional file 1: Fig. S10) is likely coupled with exon numbers, such that Pol II would take longer to release at genes with larger numbers of exons. Although defining the Pol II releasing rate in Arabidopsis remains unfeasible, our modelling result further strengthens that Pol II takes longer to terminate at genes with increased exon numbers.

## Conclusions

In this work, we provide high-resolution density maps of Ser2P Pol II in Arabidopsis under various conditions and mutant. By comprehensive data analysis combined with mathematically modelling, we found Pol II pausing at gene 3’ end is a feature innately associated with transcription termination that largely occurs before XRN3-mediated decay of cleaved readthrough transcripts. Importantly, 3’ end pause is tightly coupled with splicing at multiple exon genes, such that exon numbers by means of splicing is a major determinant of 3’ end pause level. Spliceosome assembly is required for effective 3’ end pause, while 3’ end pause promote the cooperativity of splicing among different exons at individual genes.

## Methods

### Plant materials and growth conditions

The *Arabidopsis thaliana* ecotype Columbia-0 (Col-0), and *xrn3-3* (SAIL_1172_C07) were used in this study. All seedlings used in the experiments were grown on the 1/2 MS at 22℃ (16-h light/8-h dark) for 14 days.

### pNET-seq and sequencing library construction

pNET-seq was performed in Arabidopsis seedlings as previously described with modifications [29]. In brief, approximately 2 g of seedlings was ground into fine powder with liquid nitrogen. The nuclei were extracted by nuclei lysis buffer (50 mM HEPES pH7.5, 150 mM NaCl, 1 mM EDTA, 1% (w/v) Triton X-100, 10% (w/v) glycerol, 5 mM 2-mercaptoethanol, 1 × protease inhibitor cocktail) and filtered through two layers of Miracloth. After centrifugation, the collected nuclei pellets were washed once by HBB buffer (25 mM Tris-HCl pH7.5, 0.44 M sucrose, 10 mM MgCl_2_, 0.1% (w/v) Triton X-100, 10 mM 2-mercaptoethanol) and HBC buffer (20 mM Tris-HCl pH 7.5, 352 mM sucrose, 8 mM MgCl_2_, 0.08% (w/v) Triton X-100, 8 mM 2-mercaptoethanol, 20% (w/v) glycerol) separately. Then, the nuclei pellets were resuspended in 1 ml MNase buffer (20 mM Tris-HCl pH 8.0, 5 mM NaCl, 2.5 mM CaCl_2_) and digested with 20 U MNase (TaKaRa) for 5 min at 37 °C rotating at 1400 rpm. The digested chromatin was subjected to mild sonication to release the Pol II and nascent RNA complex. After centrifuging at 15,000 rpm at 4℃ for 5 min, the supernatant was collected, followed by immunoprecipitation with anti- Ser2P CTD Pol II antibody (3E10, AB_2687450, ACTIVE MOTIF) and Protein G Dynabeads (Thermo Fisher Scientific). After immunoprecipitation, the beads were washed 8 times with washing buffer (50 mM Tris-HCL pH 7.5, 150 mM NaCl, 0.05% (v/v) NP40). The resulting nascent RNA bound by Pol II was treated with T4 PNK (Thermo Fisher Scientific) on beads for 7 min at 37 °C for RNA 5’ end phosphorylation. The resulting RNA was extracted by using TRIzol, followed by sequencing library construction with NEXTflex Small RNA-Seq Kit v3 (Bioo Scientific), according to the product manual. The sequencing library was purified with VAHTS DNA Clean Beads (Vazyme) and then used for pair-end Illumina (Nova Seq 6000, PE150) sequencing. The repeatability of sequencing data is shown in Additional file 1: Figs. S11 to S15. A summary of sequencing data is presented in Additional file 2.

### Chromatin-bound-RNA-seq (CB-RNA-seq) and sequencing library construction

CB-RNA-seq was performed as previously described [37]. Nuclei were extracted from 1 g of grinded seedlings using 8 ml Honda buffer supplemented with 1 × protease inhibitor cocktail, 2 mM DTT, 0.4 U/μl RNase inhibitor, and 200 ng/μl yeast tRNA. To enrich nuclei, the lysates were filtered through two layers of Miracloth, followed by centrifugation at 4000 rpm at 4℃ for 5 min. The resulting pellet was resuspended in 1 mL Honda buffer, followed by centrifugation at 8000* g* for 1 min. The resulting pellet was resuspended in one volume of resuspension buffer (50% (w/v) glycerol, 0.5 mM EDTA, 25 mM Tris-HCl pH7.5, 100 mM NaCl, 2 mM DTT, 1 × protease inhibitor cocktail, 500 ng/μl tRNA, 0.8 U/μl RNase inhibitor), followed by a wash with 2 volumes of washing buffer (25 mM Tris-HCl pH7.5, 300 mM NaCl, 1 M Urea, 0.5 mM EDTA, 1% (w/v) Tween 20). After centrifuging at 8000* g* at 4℃ for 1 min, an equal volume of resuspension buffer was added to resuspend the nuclei pellets again, and one volume of wash buffer was added to wash the nuclei pellets. The chromatin pellets after centrifugation were resuspended in 1 ml TRIzol, and RNA was extracted with RNA Clean & Concentrator-5 kit (Zymo Research) according to the manual. Contaminating DNA was removed using Turbo DNase (Thermo Fisher Scientific), followed by RNA purification using RNeasy Mini Kit (Qiagen). The polyadenylated RNA was removed using mRNA Capture Beads (Vazyme). The resulting RNA was treated with riboPOOLs (siTOOLs Biotech) and Streptavidin magpoly beads (Smart-Lifesciences) to remove the rRNA. The resulting chromatin-bound RNA was then used to construct the strand-specific sequencing library with NEBNext Ultra II Directional RNA Library Prep Kit (NEB). The resulting library was sent for Illumina sequencing (Nova Seq 6000, PE150). The repeatability of sequencing data is shown in Additional file 1: Figs. S11 to S15. A summary of sequencing data is presented in Additional file 2.

### RNA-seq and sequencing library construction

Total RNA was extracted using E.Z.N.A. Plant RNA Kit (Omega). DNA was removed by RQ1 RNase-Free DNase (Promega). rRNA was depleted using riboPOOLs (siTOOLs Biothch) and Streptavidin magpoly beads (Smart-Lifesciences). The resulting total RNA was used to construct the strand-specific sequencing library with NEBNext Ultra II Directional RNA Library Prep Kit (NEB) for Illumina (Nova Seq 6000, PE150) sequencing. The repeatability of sequencing data is shown in Additional file 1: Fig. S11. A summary of sequencing data is presented in Additional file 2.

### Splicing inhibitor treatment

For herboxidiene treatment, 14-day-old seedlings grown on top of a nylon filter were immersed in 50 mL 1 × PBS containing either 5 μM herboxidiene (GEX1A, Cayman Chemical) or equal amount of solvent (DMSO). The vacuum was temporarily applied and released twice to facilitate the penetration of the solution. The plants containing solution were transferred gently to an empty petri dish incubate at 22℃ in a growth chamber for 1-h treatment. The excessive solution was removed, and plants were briefly dried with kitchen paper, snap frozen in liquid nitrogen, and stored at −80 ℃ until use.

### Quantitative real-time PCR

Total RNA was extracted using E.Z.N.A. Plant RNA Kit (Omega). DNA was removed by RQ1 RNase-Free DNase (Promega). 2.5 μg RNA was used for reverse transcription (M-MLV Reverse Transcriptase, Promega) using gene-specific primers. The resulting cDNA was diluted 30 times with water. Quantitative real-time PCR was performed with a qTOWER^3^ 84 (Jena) and PerfectStart Green qPCR SuperMix (Transgen). For data normalization, intron inclusion levels are calculated by normalizing the unspliced value to the spliced value. Data are presented as mean ± standard deviation from three biological replicates.

### Total RNA-seq and CB-RNA-seq data analyses

The adapters, Ns, and low-quality bases were removed from raw data using the Trimmomatic [53] package (version 0.39), and the trimmed reads with a length less than 36 bp were also dropped. The clean reads were mapped to the TAIR10 genome using HISAT2 [54] (version 2.2.0) with default parameters. The uniquely mapped reads were retained for further processing using SAMtools [55] (version 1.3.1). The transcripts per million (TPM) value of each gene was calculated using TPMCalculator [56] (version 0.0.3). Only the expressed genes (TPM ≥ 1) were used for the calculation of the 5ʹ splicing site (5ʹSS) ratio and 3ʹ splicing site (3ʹSS) ratio, according to the method described previously [37]. The mean SS ratio of all introns was used to represent the gene’s SS ratio. The identification of differentially spliced introns was performed as described previously, except the corrected *p* < 0.05 was used as the threshold for statistical significance. To identify genes in which the intron splicing was inhibited by herboxidiene, reads at all introns or exons of a gene were summed to represent the average intron or exon levels, respectively. The values obtained from herboxidiene and DMSO samples were then used to identify the differentially spliced genes, same as for a single exon-intron unit.

### pNET-seq data analysis

The adapter was trimmed using the fastp [57] (version 0.23.1) with the parameter “--correction --overrepresentation_analysis”. Reads mapping to the reference genome and non-uniquely mapped read filtering was conducted as described above. PCR duplications were filtered by gencore [58] (version 0.16.0) according to unique molecular identifiers (UMIs). Since the 3′ end of read 1 represented the last nucleotide incorporated by the polymerase, the aligned reads were trimmed to keep only the 3′ nucleotide of read 1, with the directionality indicated by read 1. Each gene was extended 500 bp downstream of gene 3’ end to calculate a TPM value; only genes with TPM ≥ 1 were used for the calculation of the 3’ end pause index (3’PI). For metagene profiling, RPKM was calculated with a 10-bp sliding window, and profiles were visualized using plotProfile in deepTools [59].

### The calculation of 3’ end pause index

The 3’PI represents the level of Pol II at the 3’ end peak relative to its level at the gene body. Similar to the principle underlying the 5’ end pause index [29], the 3’ end pause index estimates the Pol II velocity at the 3’ end relative to the gene body region and is independent of Pol II initiation rate (or transcription level), thus can reflect the extent of Pol II pausing at 3’ end. To determine 3’PI, we first defined a terminal region for each gene as −150 to +300 bp relative to PAS. Judging from the metagene plot in Fig. 1c, Pol II often peaks around 100 bp post the PAS; therefore, ~150 to +300 bp should be sufficient to cover the Pol II 3’end pausing peak for the majority of genes. Indeed, among the 18,010 paused genes we identified, the majority of them pauses between 40 and 160 bp post the PAS, with a mean value of 97 bp post the PAS (Additional file 1: Fig. S1e). Thus, we used ~150 to +300 bp relative to PAS as definition for the termination region. In the terminal region, the pNET-seq read densities of 50-bp sliding widows (with a step size of 5 bp) were calculated, and the window closest to the PAS and with the maximum read density was selected as the proximal terminal peak. In case there are multiple windows showing equally high values, the one that is closest to the PAS was used for further analysis. 3’PI was calculated by normalizing the read density at the proximal terminal peak to the read density of the gene body region (from TSS to −301 of PAS). A gene with 3’ end pause was identified if read density in the proximal terminal region is significantly higher than that in the gene body, based on Fisher’s exact test with a Benjamini–Hochberg adjusted *p* < 0.05 [60]. Only protein-coding genes were included into the analysis. For analysis involving exon numbers, only the longest isoform from each individual gene was used to calculate the exon numbers.

### The calculation of readthrough index

The readthrough index was calculated as described previously [27] with minor modifications. The readthrough region was defined as +301 to +801 bp relative to PAS and can be extended to the TSS of a downstream gene at most. The gene body region was defined as TSS to −151 bp relative to PAS. The readthrough index was calculated as the read density of the readthrough region normalized to that of the gene body region. Fisher’s exact test (with a Benjamini–Hochberg adjusted *p* < 0.05) was used to determine whether the readthrough index was significantly different between two samples. Genes that passed the test were considered with increased or decreased readthrough levels between two samples. Only protein-coding genes were included into the analysis.

### Statistical analysis

The Spearman R value was used to measure the correlation between the two factors. The Wilcoxon test was used for any comparison between two sets of non-normally distributed data, and the *p* value was adjusted using the Benjamini–Hochberg method [60]. Fisher’s exact test was used on the overlap test between two lists and the identification of differentially paused, spliced, or readthrough genes.

### Mathematical model of Pol II occupancy

The dynamics of Pol II in initiation, elongation, and termination of gene transcription is simulated using the partial differential equation (PDE) model below.

For the genomic region between the transcription start site (TSS) and the pausing site (PS), the dynamics of Pol II is modelled using the PDE below:

$$\begin{array}{c}\frac{\partial P(x,t)}{\partial t}=F\cdot\delta \left(x\right)-\frac{\partial }{\partial x}(v\left(x\right)P\left(x,t\right))\\ \delta \left(x\right)=\left\{\begin{array}{cc}1& x=0\\ 0& x\ne 0\end{array}\right.\end{array}$$

In which $P(x,t)$ is the level of Pol II occupancy at time $t$ and genomic position with coordinate $x$. For the definition of the genomic coordinate, the site for transcription initiation is defined to have coordinate $x=0$, and positions downstream of the TSS have positive genomic coordinates. In this equation, the term $F\cdot\delta \left(x\right)$ describes the initiation of transcription which happens with a non-zero rate of initiation *F* at the TSS and zero rate at other locations. In the second term, $v\left(x\right)$ is a function that quantifies the relationship between the velocity of elongation (in other words, the movement of Pol II along the gene body). To model the pause of Pol II at the 3’ end of the gene body, we assume that the velocity of elongation decreases near the PS and the extent of velocity decrease reaches its maximum at the PS, and use the equation below to describe such a relationship between the velocity of elongation and genomic coordinate:

$$v\left(x\right)={v}_{0}\frac{{(x-{x}_{PS})}^{2}+kb}{{(x-{x}_{PS})}^{2}+b}$$

In which ${v}_{0}$ is the initial velocity of elongation when Pol II is far away from the PS, ${x}_{PS}$ is the genomic coordinate of the PS, $b$ is a parameter that quantifies the range of genomic region affected by 3’ end pause, $k$ is a parameter that quantifies the extent of velocity decrease at the PS. To simplify the model, we further assume that the initial velocity of elongation is a constant for all genes, hence ${v}_{0}=1$.

For the genomic region after the PS, we model the termination of transcription by adding a term quantifying the dissociation of Pol II from the gene:

$$\frac{\partial P(x,t)}{\partial t}=-\frac{\partial }{\partial x}\left(v\left(x\right)P\left(x,t\right)\right)-dP(x,t)$$

In which $d$ is the rate of transcription termination.

With the model developed above, we can solve the Pol II occupancy at steady state. Let $\widehat{P}(x)$ denote the level of Pol II occupancy at steady state, we have:

$$\left\{\begin{array}{l}\widehat{P}\left(x\right)=\frac{F}{v(x)}\,\,\,\,\,\,\,\,\,\,\,\,\,\,\,\,\,\,\,\,\,\,\,\,\,\,\,\,\,\,\,\,\, \mathrm{between\, TSS\, and\, PS}\\ \widehat{P}\left(x\right){v}^{\prime}\left(x\right)+v\left(x\right){\widehat{P}}^{\prime}(x)=-d\widehat{P}\left(x\right)\; \mathrm{after\, PS}\end{array}\right.$$

Hence $\widehat{P}\left(x\right)$ can be computed by directly calculating $\frac{F}{v(x)}$ for genomic region between TSS and PS or solving the ordinary differential equation $\widehat{P}\left(x\right){v}^{\prime}\left(x\right)+v\left(x\right){\widehat{P}}^{\prime}(x)=-d\widehat{P}\left(x\right)$ for genomic region downstream of PS.

### Parameter estimation of the model

Parameters in the model were determined by fitting the model to pNET-seq data obtained for different genes. To reduce noise in the experimental data, the Pol II occupancy profiles were averaged over genes with the same number of exons. In each experimental condition (e.g., Col-0, *xrn3* mutant, and so on), ten independent models were fitted separately for genes with 1, 2, 3, 4, 5, 6, 7, 8, 9, 10, or more exons using the average Pol II occupancy profiles using differential simulated annealing, a global optimization algorithm for parameter estimation of biological network models that we developed previously [61]. The objective function minimized in parameter estimation was the sum of squared error between the model-predicted and actual Pol II occupancy profiles:

$$f\left({\varvec{p}}\right)=\sum_{i=1}^{n}{\left(\widehat{P}\left({\varvec{p}},{x}_{i}\right)-{P}_{obs}\left({x}_{i}\right)\right)}^{2}$$

In which $\widehat{P}\left({\varvec{p}},{x}_{i}\right)$ is the Pol II occupancy at coordinate ${x}_{i}$ predicted by the model using parameter set ${\varvec{p}}$, ${P}_{obs}\left({x}_{i}\right)$ is the actual Pol II occupancy at coordinate ${x}_{i}$ measured using pNET-seq. For each model, the range for the coordinate $x$ was determined based on average length of genes with the corresponding exon number. The experimental data used for the fitting, ${P}_{obs}\left(x\right)$, was generated by computing the average Pol II occupancy over all genes with the same exon number. We used the average occupancy profiles to fit the model to reduce noises in the pNET-seq data and improve the robustness of the model. After the parameter set ${{\varvec{p}}}_{opt}$ with optimal fitting was found for each model, we first computed the optimal value of the objective function, ${f}_{opt}=f({{\varvec{p}}}_{opt})$, and then sampled 50,000 random parameter sets in the region $[0.316{{\varvec{p}}}_{opt},\, 3.16{{\varvec{p}}}_{opt}]$ using Latin hypercube sampling and kept all parameter sets ${\varvec{p}}$ with $f\left({\varvec{p}}\right)<2{f}_{opt}$ for the following calculation of 3’ end pausing time.

### Calculation of 3’ end pausing time

As mentioned in the main text, the 3’ end pausing time was defined as the increase in total time required for completion of transcription in the scenario with 3’ end pause compared to the scenario without 3’ end pause.

For the scenario with 3’ end pause, the total time for the entire process of transcription initiation, elongation, and termination to complete was computed as below:

$${t}_{0}={\int }_{0}^{{x}_{PS}+1000}\frac{dx}{v(x)}={\int }_{0}^{{x}_{PS}+1000}\frac{{(x-{x}_{PS})}^{2}+b}{{(x-{x}_{PS})}^{2}+kb}dx$$

For the scenario without 3’ end pause, the velocity of elongation always equals ${v}_{0}=1$, hence the total time required for the completion of transcription is:

$${t}_{1}={x}_{PS}+1000$$

Thereby, the 3’ end pausing time is calculated using the equation below:

$${t}_{\mathrm{pause}}={t}_{0}-{t}_{1}$$

### Supplementary Information

**Additional file 1: Fig. S1-S15.** Supplementary figures.

**Additional file 2: Table S1.** Summary of sequencing data.

**Additional file 3.** Review history.

## Data Availability

The raw sequencing data and processed files for pNET-seq, CB-RNA-seq, and total RNA-seq data generated in this study are available in the Gene Expression Omnibus (GEO) database under accession number GSE205545 [62]. For pNET-seq of Ser 2P (CMA602), Ser 5P (CMA603), and Unph (8WG16) were acquired from published studies (GSE109974 [63] and GSE117014 [64]). The python scripts used for Pol II pause, RNA readthrough, and SS ratio in this study are available under the MIT license at GitHub repository: https://github.com/flzh628/Pol_II_pause [65] and at Zenodo: https://zenodo.org/record/8287036 [66]. The processed single base pair.bw files of pNET-seq data for each biological repeat are available under the MIT license at GitHub repository: https://github.com/flzh628/Pol_II_pause/tree/main/pNET-seq_sample_bw_files [67]. MATLAB scripts implementing the mathematical model are available under the MIT license at the GitHub repository: https://github.com/ziweidai/pol_II_pause [68] and at Zenodo: https://zenodo.org/record/8296364 [69].
