# Supplementary material for: Coupling of co-transcriptional splicing and 3’ end Pol II pausing during termination in Arabidopsis
Source: Genome Biol. 2023 Sep 11;24:206. doi: 10.1186/s13059-023-03050-4 (PMC10496290; doi:10.1186/s13059-023-03050-4)
Supplement: Supplementary file 1 — Additional file 1: Fig. S1-S15. Supplementary figures. [file 13059_2023_3050_MOESM1_ESM.pdf]

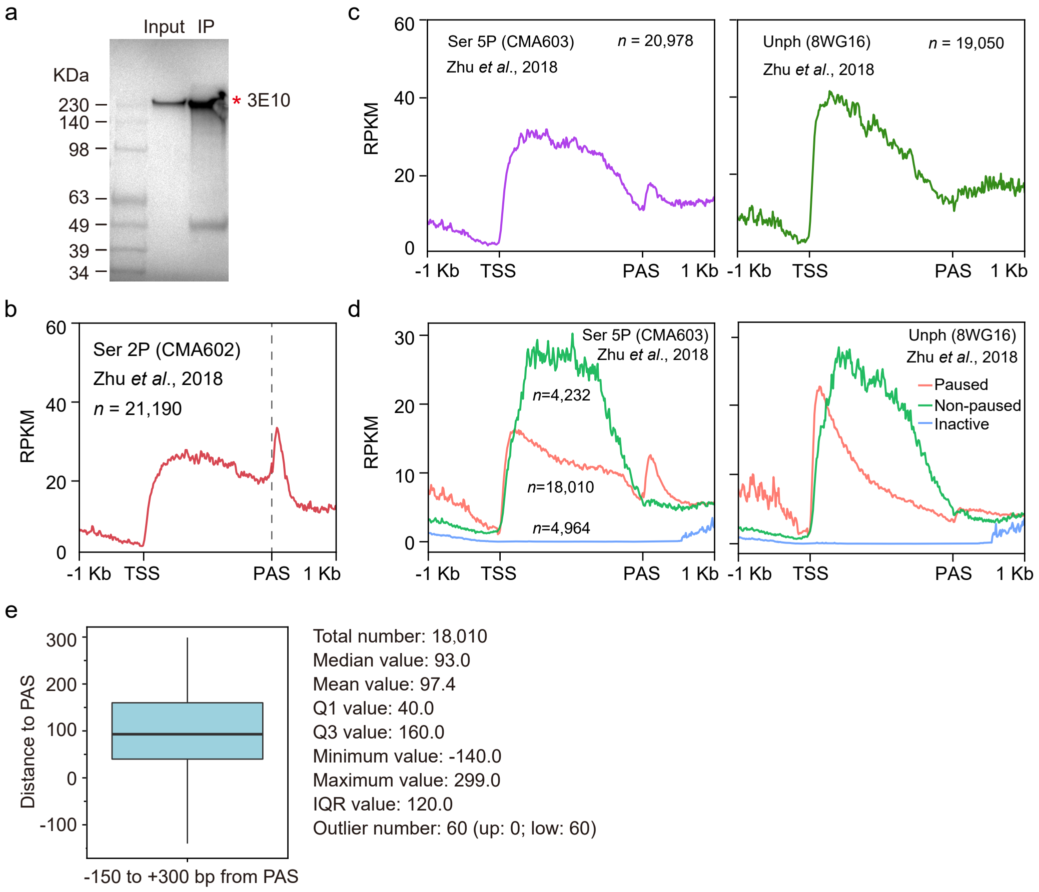

Fig. S1 Profiles of Pol II with different modifications enriched at its C-terminal domain. a Western blot showing the immunoprecipitated Ser2P Pol II from Arabidopsis nuclei extracts using 3E10 antibody. **b** Metagene plot showing the Ser2P Pol II profile generated by pNET-seq with CMA602 antibody as published previously. Only the expressed protein-coding genes (TPM≥1 based on the Ser2P Pol II pNET-seq data) are used for analysis [29]. **c** Metagene plot showing the Pol II profiles generated by pNET-seq with different antibodies as published previously. Only the expressed protein-coding genes (TPM ≥1 based on the Ser2P Pol II pNET-seq data) are used for analysis [29]. **d** Profiles of Pol II at paused genes, non-paused genes, and inactive genes in a previously published data set. 3' end pausing state of genes is defined by Ser2P pNET-seq data in this study, as shown in Fig. 1c. **e** Distribution of the Pol II pausing position downstream of PAS. 18,010 paused genes are used for analysis. For box plots, the line indicates the median, box edges represent the first and third quartiles, and the whiskers extend to the farthest data points within 1.5 × interquartile range outside box edges.

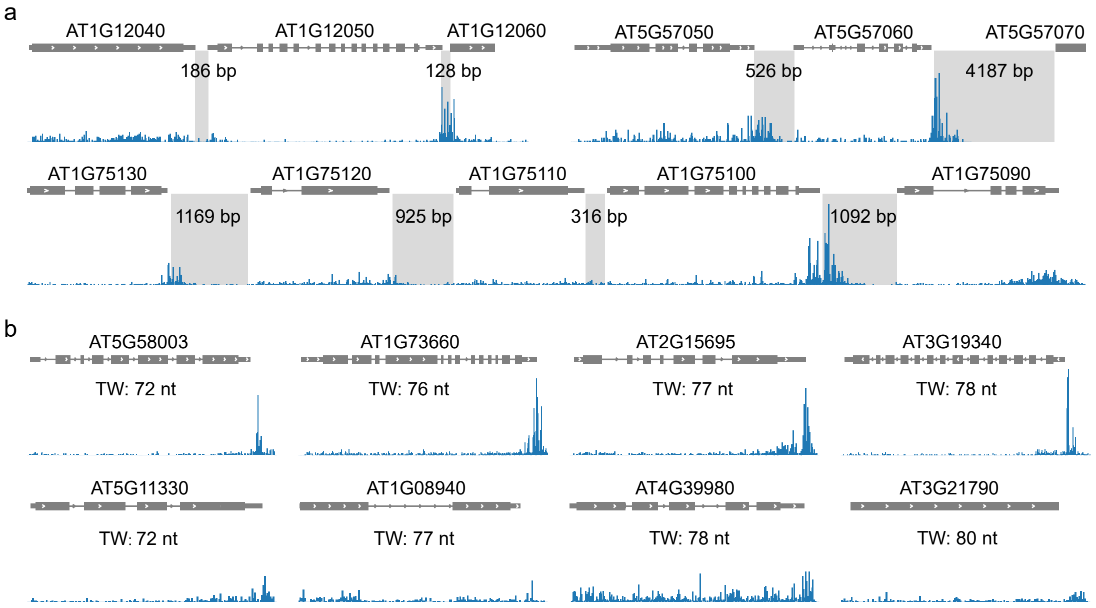

Fig. S2 Relationship between 3' end Pol II pause and the intergenic region length or termination window size. a Example of genes showing the levels of 3' end pause is not associated with the length of the intergenic region. The grey bars indicate the distance from the PAS of the upstream gene to the TSS of the downstream gene. **b** Example of genes with small termination window size (TWS) showing either a high level of 3' end pause (top panel) or a low level of 3' end pause (bottom panel). TWS of each gene is indicated below the gene structure.

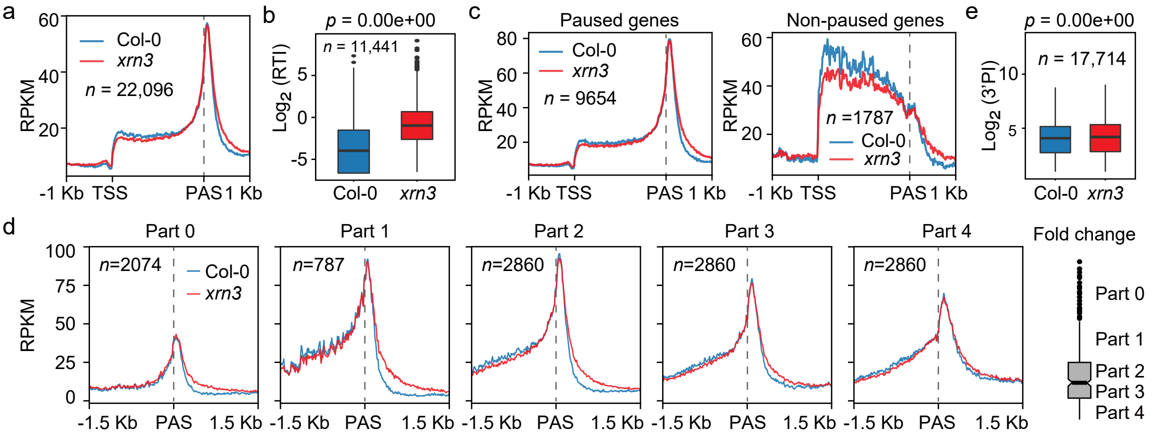

Fig. S3 The impact of XRN3 on 3' end Pol II pause. **a** Metagene plot showing Ser2P Pol II profile in Col-0 and *xrn3* of all the expressed protein-coding genes (22,096). **b** Box plot comparing the readthrough index (RTI) between Col-0 and *xrn3* for genes that RTI were up-regulated in *xrn3*. *p* value was calculated based on a paired Wilcoxon test. **c** Metagene plots showing Pol II profiles in Col-0 and *xrn3* of paused and non-paused genes which display significantly increased RTI in *xrn3* compared with Col-0 as shown in Fig. 2c. Paused and non-paused genes were defined based on the 3'PI in Col-0. **d** Metagene plot showing Pol II profiles in Col-0 and *xrn3* of genes which displayed significantly increased RTI in *xrn3* compared with Col-0. Genes were divided into five groups according to the fold change of RTI in *xrn3* compared with Col-0 (Part 0 to Part 4, from high to low). **e** Box plot comparing 3'PI in Col-0 and *xrn3* at 17,714 protein genes that are paused in both Col-0 and *xrn3*. *p* value was calculated based on a paired Wilcoxon test.

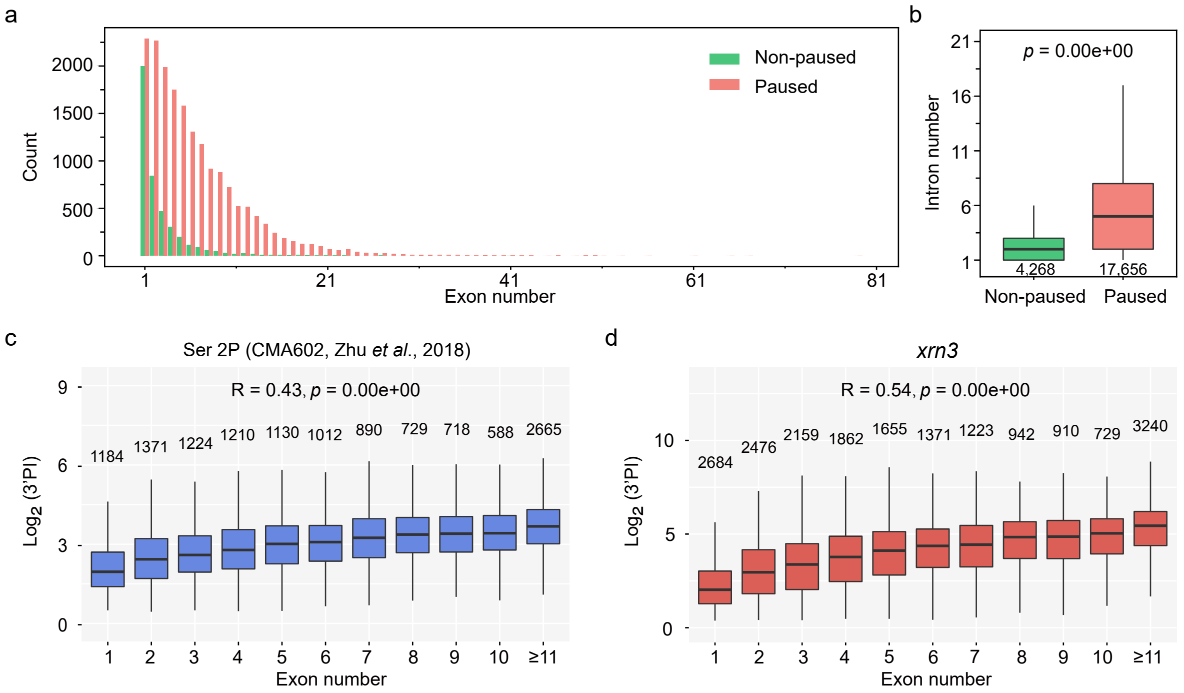

Fig. S4 3' PI is associated with exon numbers. **a** Distribution of gene exon numbers of paused and non-paused genes in Arabidopsis genome. The same gene sets as Fig. 1c are used. **b** Box plot comparison of gene exon numbers between paused and non-paused genes. Genes with less than 20 introns are plotted. *p* value was calculated based on a Wilcoxon test. **c** The relationship between 3'PI and intron numbers based on Ser2P pNET-seq data published previously[29]. **d** The relationship between 3'PI and exon numbers in *xrn3*. For **c** and **d**, the Spearman correlation coefficient is indicated on top of the plot. For box plot, gene numbers of each group are shown above the boxes.

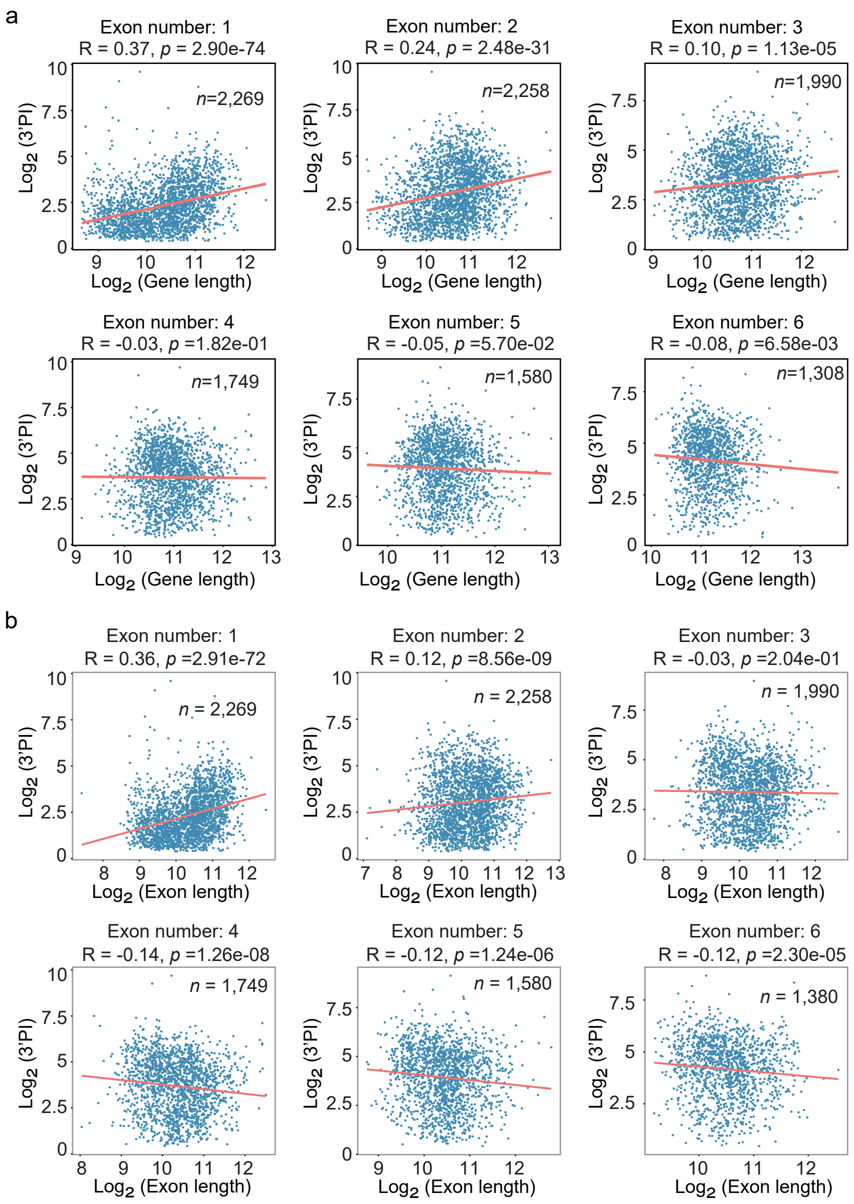

Fig. S5 Relationships between 3'PI and gene length or exon length in gene groups with fixed numbers of exons. a Scatter plots indicating the relationship between 3'PI and gene length in gene groups with fixed exon numbers. **b** Scatter plots indicating the relationship between 3'PI and the sum of exon length in gene groups with fixed exon numbers. For each panel, the orange line indicates the trendline fitted based on the least squares method. Spearman correlation coefficient is indicated on top of the plot.

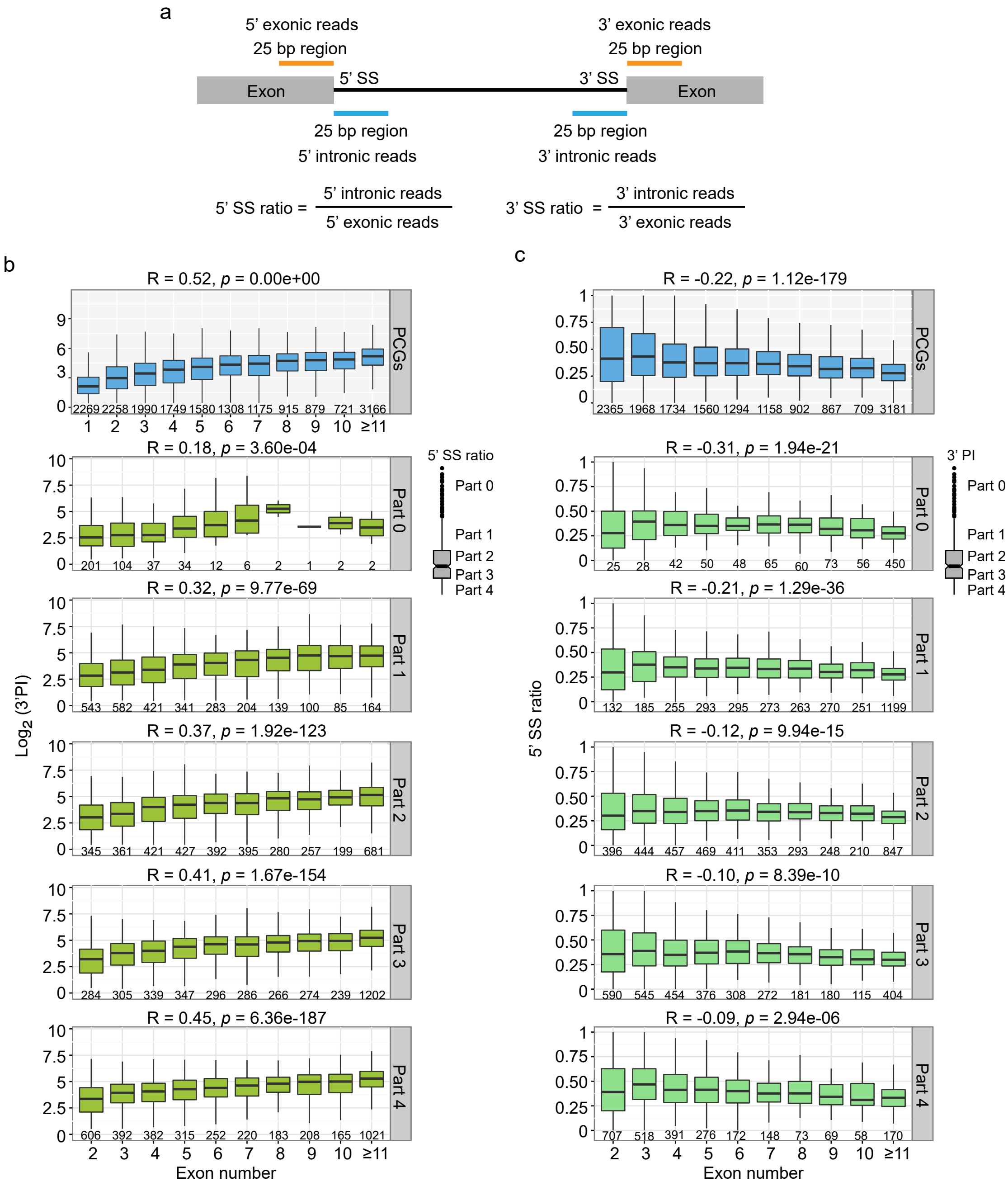

Fig. S6 Relationships among exon numbers, 3' end pause and splicing efficiency. **a** Diagram showing the definition of 5'SS ratio and 3'SS ratio. Note that the SS ratios reflect intron retention levels. **b** Box plots demonstrate the relationship between 3'PI and exon numbers in gene groups with different levels of 5'SS ratio. The plot in blue demonstrates the situation in all protein-coding genes. Protein-coding genes were divided into five groups according to average 5'SS ratios of genes (part 0 to part 4, from high to low), and the relationship between 3'PI and exon numbers was plotted separately for each group. **c** Box plots demonstrate the relationship between the 5'SS ratio and exon numbers in gene groups with different levels of 3'PI. The plot in blue demonstrates the situation of all protein-coding genes. Protein-coding genes were divided into five groups according to the 3'PI (part 0 to part 4, from high to low), and the relationship between the 5'SS ratio and exon numbers was plotted separately for each group. For **b** and **c**, The Spearman correlation coefficient is indicated above each plot. Gene numbers of each group were shown below the boxes.

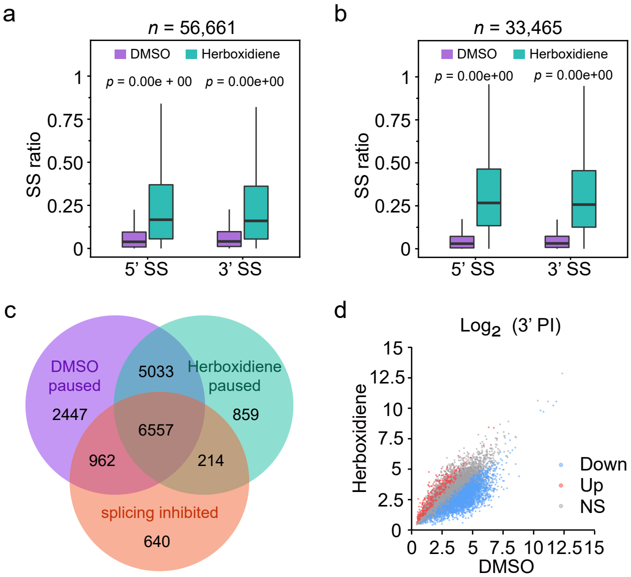

Fig. S7 Inhibition of splicing reduces 3'PI. a Box plot showing 5'SS ratios and 3'SS ratios of 56,661 exon-intron-exon units that display total RNA-seq reads in the Arabidopsis genome. **b** 5' and 3'SS ratios of 33,465 exon-intron-exon units, of which splicing was inhibited after herboxidiene treatment. For **a** and **b**, the *p* value was calculated based on a paired Wilcoxon test. **c** Overlaps among paused genes in DMSO treated sample, paused genes in herboxidiene treated sample and genes that displayed splicing inhibition upon herboxidiene treatment. **d** Scatter plot demonstrating that 3'PI is generally reduced after herboxidiene treatment compared with DMSO control. Genes with up or down-regulated 3'PI were determined based on Fisher's exact test of statistical difference ($p < 0.05$).

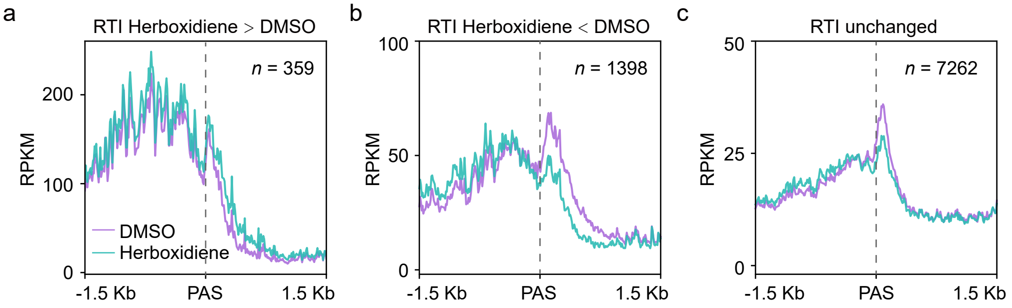

Fig. S8 The impact of splicing inhibition on transcription readthrough. Metagene plot showing Pol II profiles at gene 3' end in DMSO control group and herboxidiene treated group. **a** Genes of which readthrough levels were up-regulated by herboxidiene treatment compared with DMSO control. **b** Genes of which readthrough levels were down-regulated by herboxidiene treatment compared with DMSO control. **c** Genes of which readthrough levels were unchanged by herboxidiene treatment compared with DMSO control.

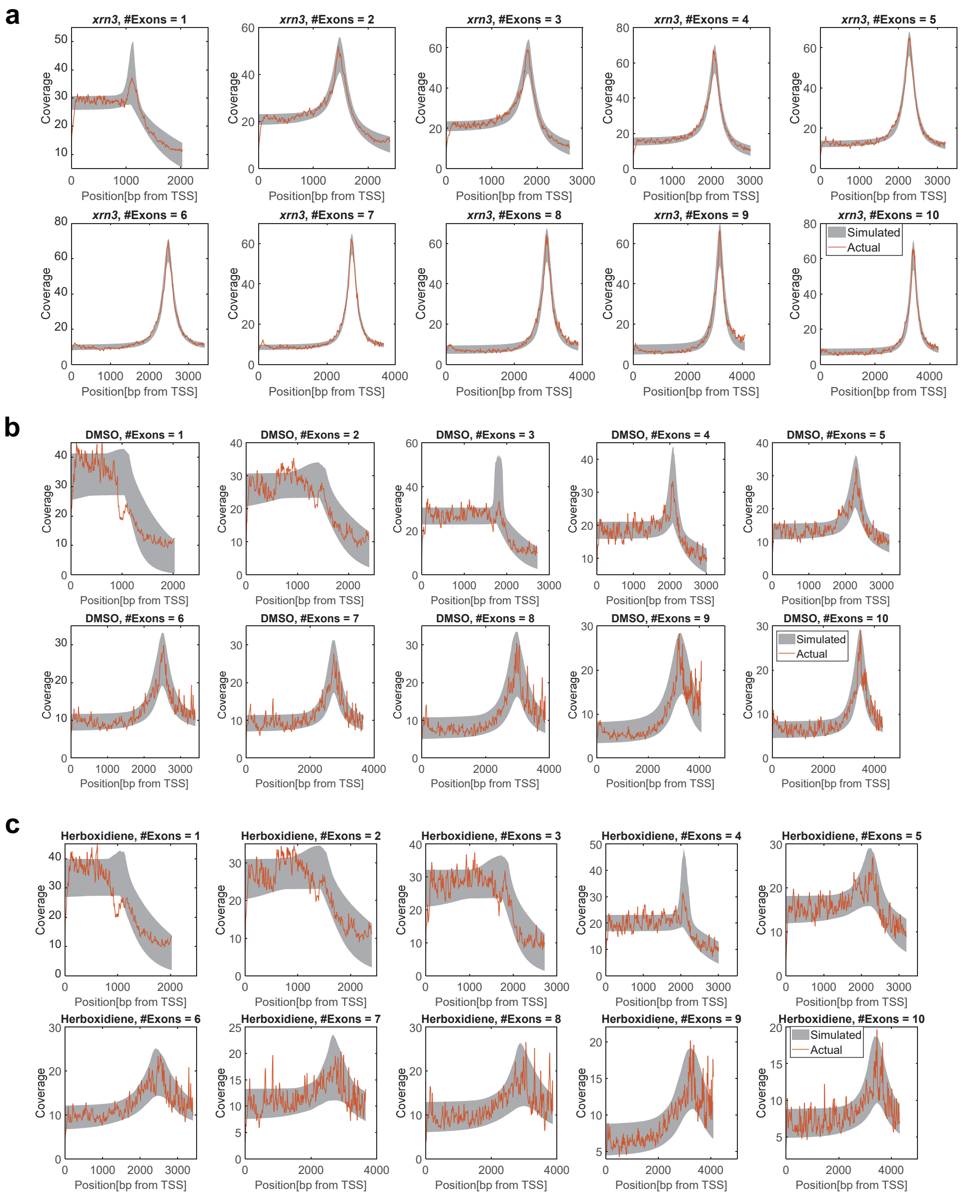

Fig. S9 Comparison of model-predicted and actual Pol II occupancy profiles in a *xrn3*, **b** DMSO, and **c** Herboxidiene.

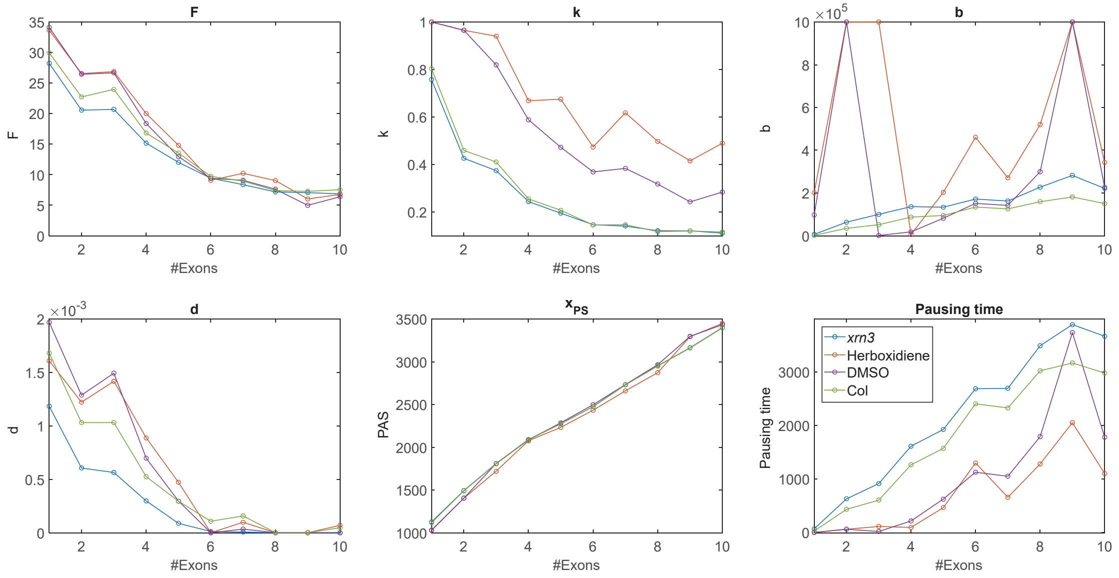

Fig. S10 Model parameters that fit the pNET-seq data the best in different genes and experimental groups.

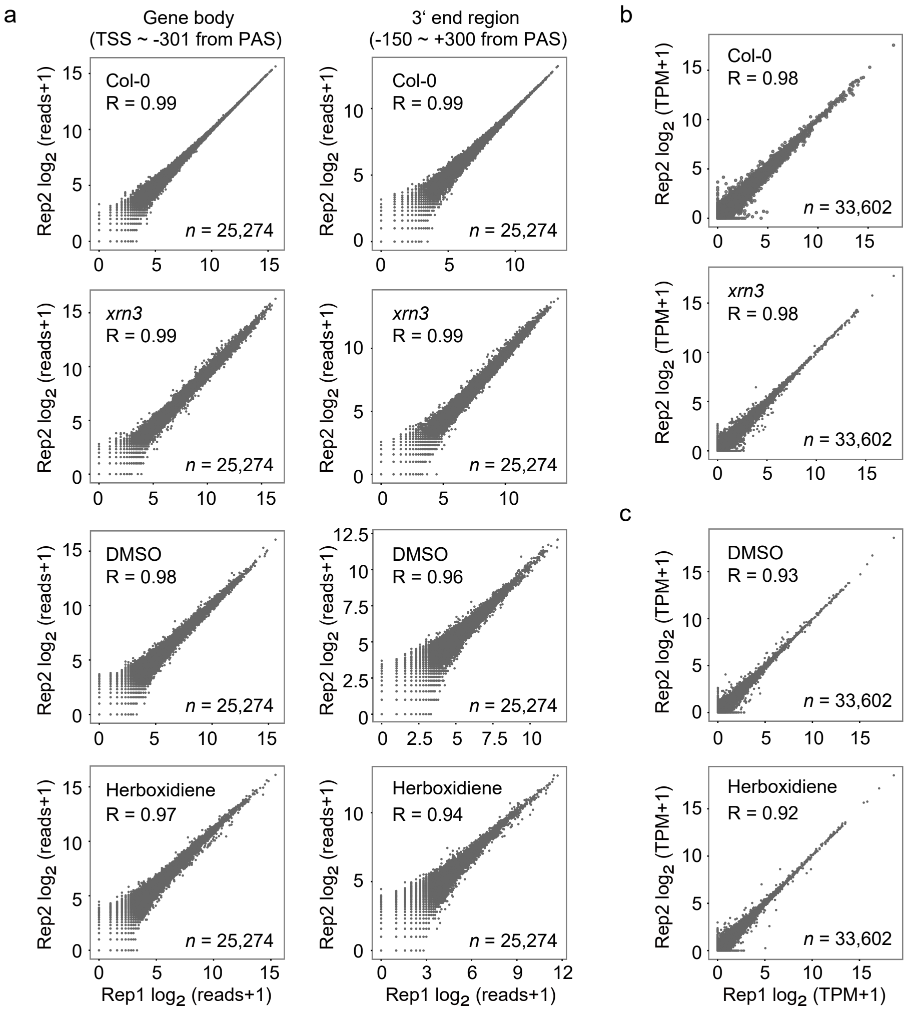

Fig. S11 Summary of sequencing data quality. **a** Scatter plot indicating the correlation between biological replicates of pNET-seq reads at gene body (left panel) and 3' end region (right panel). Spearman R is shown. **b**, **c** Scatter plots indicating the correlation between biological replicates of CB-RNA-seq and total RNA-seq, respectively. Spearman R is shown.

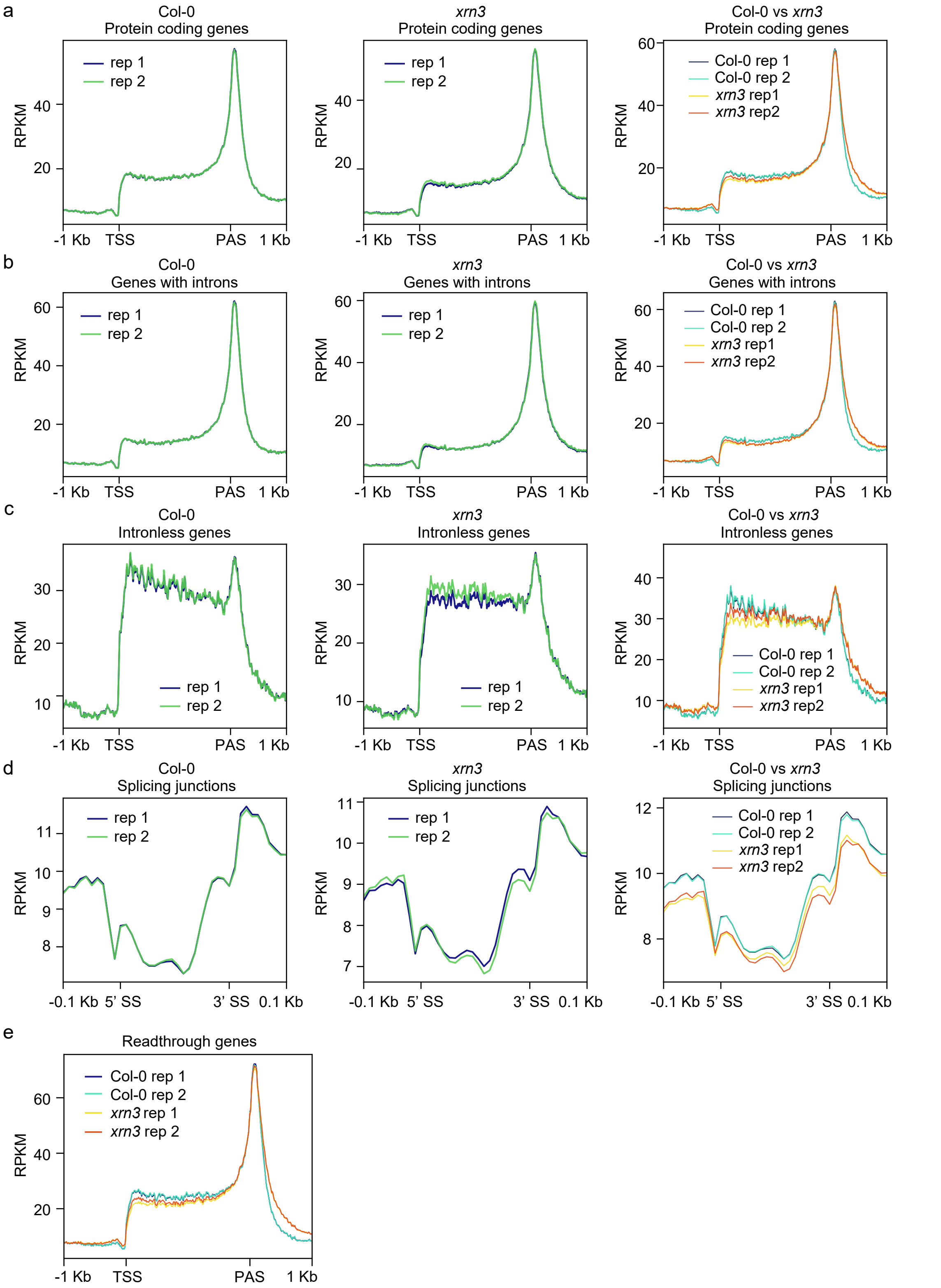
a
Col-0
Protein coding genes
xrn3
Protein coding genes
Col-0 vs xrn3
Protein coding genes
b
Col-0
Genes with introns
xrn3
Genes with introns
Col-0 vs xrn3
Genes with introns
c
Col-0
Intronless genes
xrn3
Intronless genes
Col-0 vs xrn3
Intronless genes
d
Col-0
Splicing junctions
xrn3
Splicing junctions
Col-0 vs xrn3
Splicing junctions
e
Readthrough genes
rep 1
rep 2
Col-0 rep 1
Col-0 rep 2
xrn3 rep1
xrn3 rep2
xrn3 rep 1
xrn3 rep 2
RPKM
-1 Kb
TSS
PAS
1 Kb
-0.1 Kb
5' SS
3' SS
0.1 Kb

Fig. S12 Metagene plots of pNET-seq data from different biological replicates of Col-0 and *xrn3*. Metagene plots demonstrate the distribution of Ser2P Pol II of different gene groups along gene features based on pNET-seq data. Results from each biological replicate were plotted separately. **a** Results from all the protein-coding genes. **b** Results from genes with introns. **c** Results from the intronless genes. **d** Metagene profile at the splicing junctions (exon-intron-exon units). **e** Results from the genes that display significantly increased RTI in *xrn3* compared with Col-0.

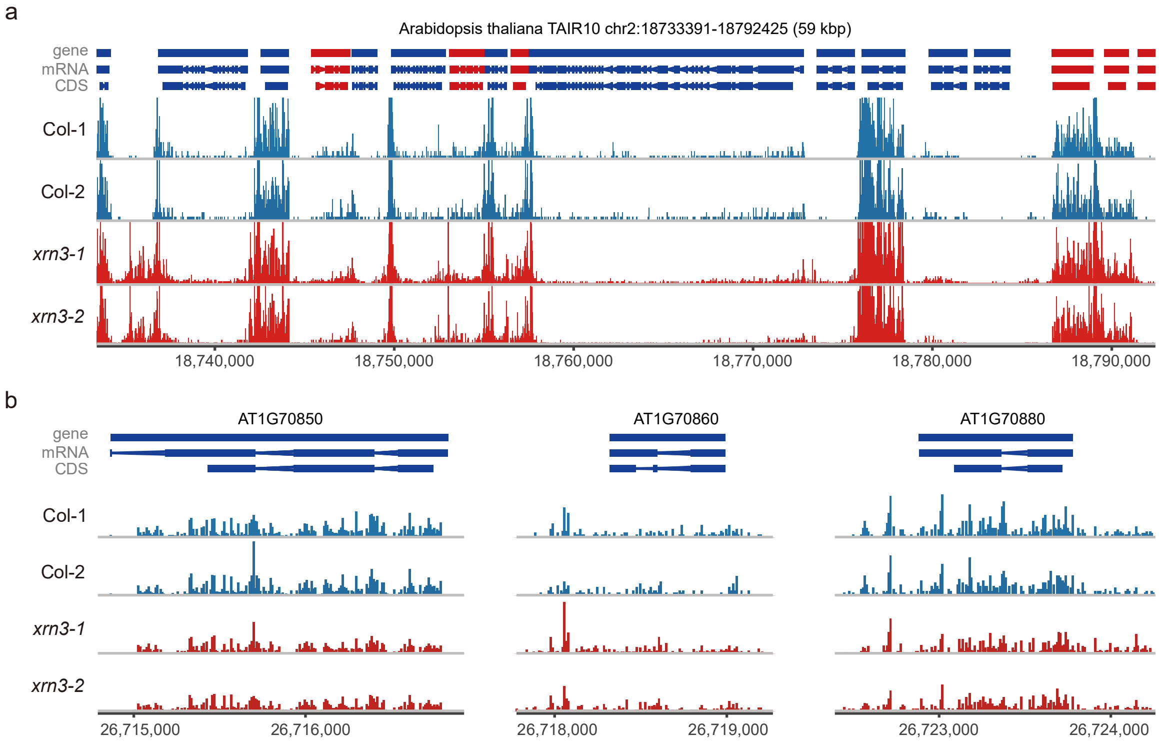

Fig. S13 Genome browser tracks of pNET-seq data from different biological replicates of Col-0 and *xrn3*. **a** Genome browser tracks of a 59 kbp window from chromosome 2. **b** Genome browser tracks of 3 randomly selected genes from chromosome 1.

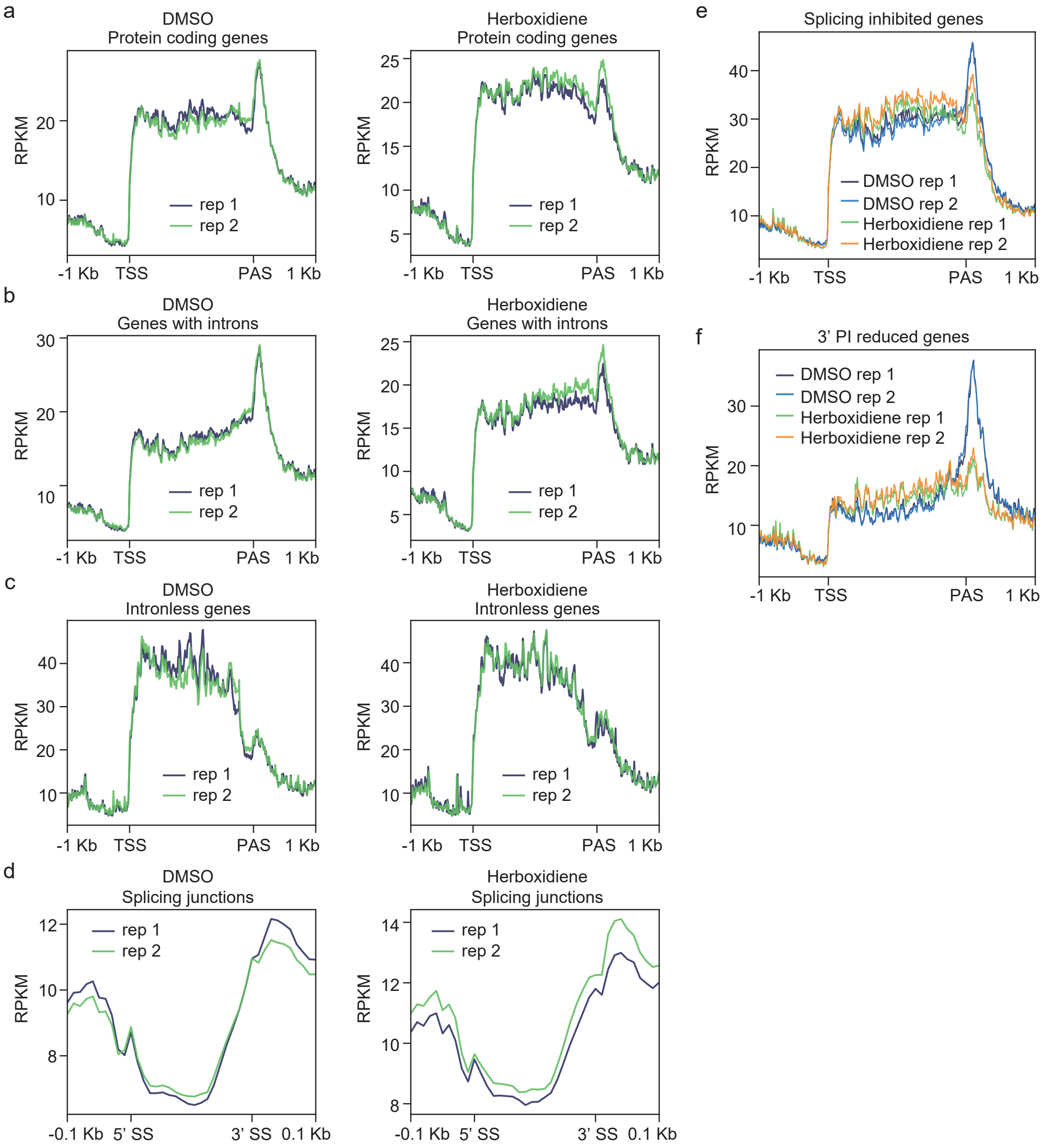

Fig. S14 Metagene plots of pNET-seq data from different biological replicates of Col-0 treated with DMSO or herboxidiene. **a** Results from all the protein-coding genes. **b** Results from genes with introns. **c** Results from the intronless genes were plotted. **d** Metagene profile at the splicing junctions (exon-intron-exon units). **e** Results from the genes that display splicing inhibition upon herboxidiene treatment. **f** Results from the genes that displayed significantly reduced 3'PI upon herboxidiene treatment.

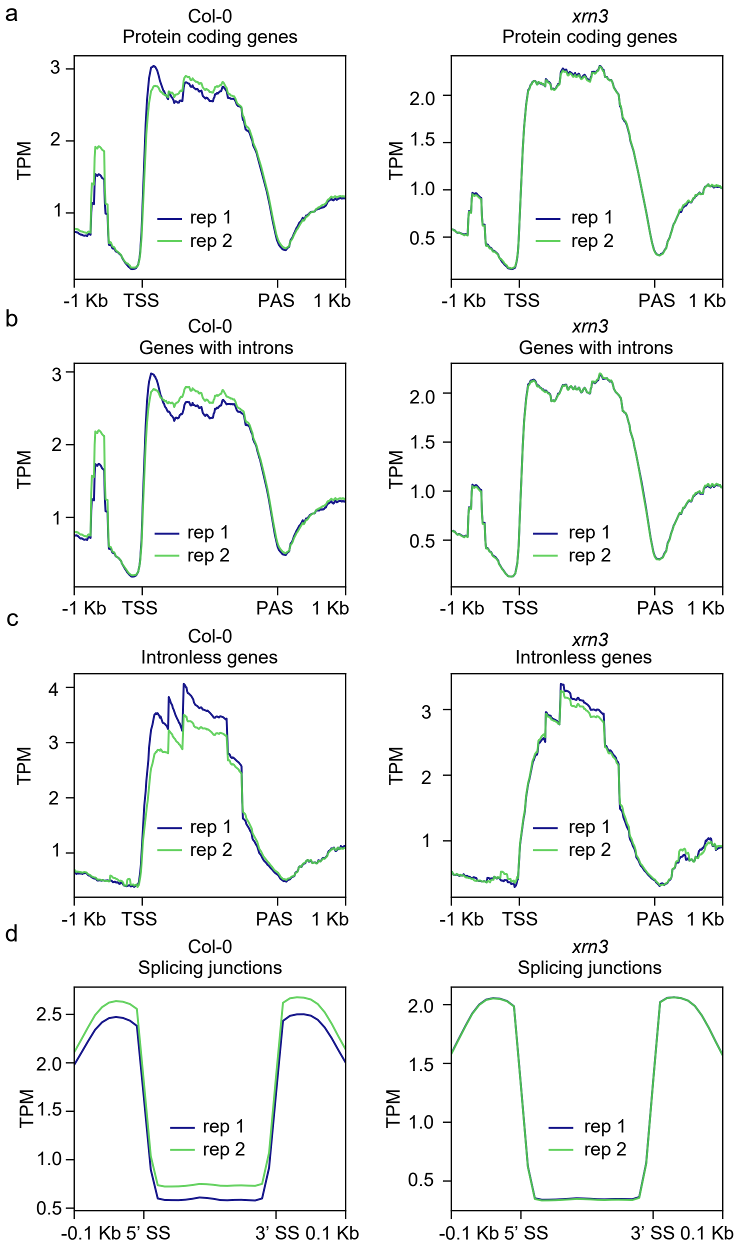

Fig. S15 Metagene plots of CB-RNA-seq data from different biological replicates of Col-0 and *xrn3*. Metagene plots demonstrate the distribution of nascent RNA from different gene groups along gene features based on CB-RNA-seq data. **a** Results from all the protein-coding genes. **b** Results from genes with introns. **c** Results from the intronless genes. **d** Metagene profile at the splicing junctions (exon-intron-exon units).
