# Supplementary material for: Coupling of co-transcriptional splicing and 3’ end Pol II pausing during termination in Arabidopsis
Source: Genome Biol. 2023 Sep 11;24:206. doi: 10.1186/s13059-023-03050-4 (PMC10496290; doi:10.1186/s13059-023-03050-4)
Supplement: Supplementary file 3 — Additional file 3. Review history. [file 13059_2023_3050_MOESM3_ESM.docx]

Review History

**First round of review**

**Reviewer 1**

**Were you able to assess all statistics in the manuscript, including the appropriateness of statistical tests used?**

No, I do not feel adequately qualified to assess the statistics.

**Comments to author:**

This is an interesting manuscript that deals with the connection of splicing and 3' end Pol II pausing. The authors has used a range of mutants and methods to show that Pol II pausing at genes 3' end in Arabidopsis is observed predominantly at genes with introns and that splicing somehow contributes to this phenomena.

I like the work and fell that the main conclusion is justified. I have some reservation to the way authors present quality controls for their experiments. While I liked the logical flow in the paper I would very much like to see the controls in the supplement including quality controls for the pNETseq and CB-RNA-seq. I would like to ask for meta profiles for pNET and CB-RNA for whole gene, and splicing junctions for all genes, genes with no introns, genes with introns, genes with readthrough in fcafpa, readthrough in xrn and changed splicing after herboxidiene treatment as well as genes with changed pausing in herboxidiene.

Regarding figure 4 I would also like to ask the authors to repeat this analysis with only exon sequence considered. to make sure the change is not because of "dilution" of exonic sequence with intronic contribution.

Minor points:

#140 is the word density here use in its true meaning?- (gene length independent ?)

when taking about nanopore (TWS) data lack of corelation with pausing, The authors may consider an alternative explanation- different stability of readout?

Is readthrough index length normalized ( line 178) ?

Fig2c is this data based on planet-seq? If so then maybe one can not say XRN do not control pausing as peak is broader?

I would like the author to discuss the fact that xrn3-3 used by them is a weak allele and xrn3-8 or fry would be more suitable here.

Finally the herboxidiene used by the authors to stop the splicing has been used before in plants for the same purpose (Dolata et al 2015). Interestingly authors, reported no effect of herboxidiene on local Pol II elongation at the splice sites with is consistent with the effect of herboxidiene on splicing that feeds on the 3' pausing as reported here.

**Reviewer 2**

**Were you able to assess all statistics in the manuscript, including the appropriateness of statistical tests used?**

Yes, and I have assessed the statistics in my report.

**Comments to author:**

The authors generated Ser2P Pol II positioning data specifically enriched at gene 3' end and defined the 3' end pause index (3'PI) to group genes with or without 3' end pausing. They also incorporated published datasets into their analysis with similar or different treatments. They concluded that the number of exons is a major determinant of Pol II pause and proposed a model to estimate pausing time for genes with different exon numbers.

Major points,

1. The interpretation of pNET-seq need to be improved significantly.

1a, As the major method used in this manuscript, the introduction for pNET-seq (Line 96-100) is hard to follow.

Line 99, The word "peak" appeared suddenly without stating anything related to immunoprecipitation.

Line 101-103, Pol II with Ser5 phosphorylated CTD is related to spliceosome but can not be simply proved by the pNET-seq result using total and Ser5P specific Pol II antibodies.

Line 102, Is this "NET-seq" different from pNET-seq?

Line 105 Please try to re-write "Ser2P Pol II is mainly associated with active elongation and Pol II…"

Line 106, Please at least include Pol II CTD with Ser2 phosphorylation.

Line 107, the upper case FLC usually means a protein.

Line 107-110, The mammalian genomes are usually 20 times larger than Arabidopsis, and the average transcript length is also 8-10 times. What did the author want to say by comparing "a few thousand base pair " in mammals to "300 bp" in Arabidopsis? "300 bp" too long or too short?

Line 111, The median size of the termination window detected using nascent RNA sequencing is 161 nt. Why this size coincides with "300 bp"?

Line 114, which Pol II peak? Ser2P/Ser5P or total? Why BORDER is "Consistently" with the rest part of the paragraph?

1b, Line 121, the choices for antibodies for Pol II CTD should be an important part for pNET-seq.

These monoclonal antibodies are designed for mammals. What is the specificity of this antibody (3E10) in plants? Why do the authors prefer 3E10 over CMA602? The selection of antibodies needs to be mentioned in the introduction.

Moreover, what is the rationale for re-do an experiment using a different antibody other than specificity? Could the difference between the previous publication (ref39) and the current study fall into the confidential interval between replicates? For example, what is the variation between two IP experiments using the same antibody? The author needs to provide replicates to claim the difference.

2. The correlation analysis for figure 1 is confusing. What is the cutoff for a significant correlation? The P values are all very small though some of the figures seem to be just random.

3. The analysis for readthrough transcript and 3' paused transcript is interesting. Can the author calculate and compare the proportion of paused/readthrough from pNET-seq or CB-RNA-seq for better comparison?

The significance for I would also suggest performing paired comparisons between same genes in Col-0 and fca/fpa mutant instead of using tests for independent variables.

Line 206, The significace between 3'PI index seems mild. And the trend for 3'PI change in all genes and the genes with increased readthrough is the same (Figure 3 and S4), which means the increased readthrough caused by fca fpa mutant has no impact on 3' end pause?

4. The conclusion of 3' end Pol II pause correlates with the number of introns is a tricky part.

The gene length itself still seems to be a better indicator. First, genes has isoforms, the total gene number of all bars in figure 4 c&d is far larger than the gene number (18,038) in figure 1e, indicating that they are using isoforms in figure 3c&d, but not in figure 4e. Second, the correlation analysis in figure S5e also indicates a good correlation in 3'PI and gene length with intron number <=3. And the authors may need to change the correlation analysis in figure 5e from categories to number, which is used in figure 4e.

The splicing defect mutants or the herboxidiene treatment could differentiate the effect of the intron/exon number with gene/isoform length. However, the intron retention also increased the length of the transcript.

5. If the author admit that the gene length rather than intron number is the key to Pol II pausing, the modelling of Pol II occupancy could be simplified by incorporating a decrease of elongation speed alongside the gene. And why the authors have to fit 10 models for genes with different intron numbers?

Minor points:

Line 26, please rewrite "3'PI is a function of gene intron numbers and co-transcriptional splicing efficiency"

Line 93 PacBio

Figure 4f, I would suggest putting the count number on top.

Line 211, Usually we would use the number of exons instead of introns.

**Authors Response**

**Point-by-point responses to the reviewers’ comments:**

*We thank both reviewers for their constructive feedback, which has offered the opportunities for us to improve our manuscript. We have revised our manuscript carefully according to the comments and suggestions from reviewers. Below is our point-by-point response to the reviewer’s comments.*

Reviewer #1:

This is an interesting manuscript that deals with the connection of splicing and 3' end Pol II pausing. The authors has used a range of mutants and methods to show that Pol II pausing at genes 3' end in Arabidopsis is observed predominantly at genes with introns and that splicing somehow contributes to this phenomena.

I like the work and fell that the main conclusion is justified. I have some reservation to the way authors present quality controls for their experiments. While I liked the logical flow in the paper I would very much like to see the controls in the supplement including quality controls for the pNETseq and CB-RNA-seq. I would like to ask for meta profiles for pNET and CB-RNA for whole gene, and splicing junctions for all genes, genes with no introns, genes with introns, genes with readthrough in fcafpa, readthrough in xrn and changed splicing after herboxidiene treatment as well as genes with changed pausing in herboxidiene.

*Response: We thank reviewer 1 for constructive comments and many valuable suggestions. We have added the analysis of pNET-seq and CB-RNA-seq for meta-profiles of different gene sets as the reviewer suggested. The results can be found in the updated Fig. S13, S14 and S15. These profiles revealed the expected patterns and in general good reproducibility between biological replicates. We hope we have addressed the reviewer’s concern here.*

Regarding figure 4 I would also like to ask the authors to repeat this analysis with only exon sequence considered. to make sure the change is not because of "dilution" of exonic sequence with intronic contribution.

*Response: We thank the reviewer for raising this point. We reasoned that the relationship among intron, exon and 3’PI can be dissolved from the following aspects. Firstly. intron number is bound with exon number, and therefore both of them are associated with gene length and 3’PI. Secondly, as suggested by the reviewer, we have added an analysis (see figure below, and revised Fig. S6b, updated main text line 242-248) where we looked the relationship between exon length and 3’PI when the exon or intron numbers are fixed. In this case, as the reviewer mentioned, only the exon sequence is considered. The results showed that exon length does not correlate positively with 3’PI, except for the single-exon genes. For genes that have more than 4 exons, the exon length seems even mildly correlated with 3’PI in a negative way. Overall, no definitive pattern can be observed. Thus, taking into account the above result and the results showed in Fig. 4 and Fig. S6a, our conclusion that the intron or exon numbers is a major contributing factor in determining the 3’pause remains solid.*

*Figure (updated Fig. S6b). The relationship between 3’PI and exon length at gene groups with fixed numbers of exons. To exclude the potential impact from the intron or exon numbers, genes are divided into groups based on their exon numbers.*

Minor points:
#140 is the word density here use in its true meaning?- (gene length independent ?)

*Response: We apologize for not describing the word “density” clearly. Generally, the word “density” in our manuscript means the abundance of Pol II per unit length, which is calculated by the read count of pNET-seq in the indicated region divided by the length of the region. For the issue at #140, the original sentence is “ Of note, the density of Pol II is determined by Pol II initiation rate over its elongation rate”. To increase clarity, we have changed it into the following “Of note, the level of Pol II at any defined position of a gene is determined by Pol II initiation rate over its elongation rate at that position”. We hope it is clearer now.*

when taking about nanopore (TWS) data lack of corelation with pausing, The authors may consider an alternative explanation- different stability of readout?

*Response: We appreciate this comment. First, we would like to state that there is actually consistency between our results and the previous nanopore results. As shown in the below figure, Mo et al (Genome Biology 2021) showed that the end of the termination window (TWE) can be aligned well with the 3’ Pol II peak as detected in pNET-seq or Pol II ChIP-seq data. This is consistent with our result in Fig. 1e, that the distance from the 3’ end Pol II peak to poly (A) site is correlated with termination window size.*

*In the meanwhile, our results showed that the extent of pausing (3’PI )does not correlate strongly to the termination window size (Fig. 1f). This result is reasonable given that we find the intron numbers is a major contributor of 3’ end pause. We appreciate the reviewer raising the possibility that there might be different sensitivity in terms of the readout from the pNET-seq data and the nanopore data. Currently, we reasoned this is unlikely the major reason for the lack of correlation between TWS and 3’PI. Nanopore data detect the change of TWS very sensitively such that it is varying among individual genes with different termination efficiency ^1^(Mo et al, Genome Biology, 2021). 3’PI is also detected robustly in our pNET-seq data such that it is tightly associated with the intron numbers at individual genes. In light of the reviewer’s comments, we have edited the text (see below) to state more clearly that our result is generally not in conflict with but consistent with previous observations.*

*“Consistent with previous observations, the position of 3’ end pause is well-matched with TWS such that the bigger the TWS, the longer distance between 3’ end pause and poly (A) site can be observed (Fig. 1e).” – updated main text line 171-173.*

Is readthrough index length normalized ( line 178) ?

*Response: Yes, the readthrough index (RTI) is length normalized, the calculation of RTI is shown in Fig. 2b.*

Fig2c  is this data based on planet-seq? If so then maybe one can not say XRN do not control pausing as peak is broader?

*Response: Thanks for the comment. In our manuscript, we used pNET-seq with Ser2P antibody to generate Fig. 2c. Genes with significantly increased readthrough in xrn3 compared with Col-0 were analyzed in Fig.2c. The peak is broader in xrn3 than in Col-0 because of the increased readthrough transcript after the poly (A) site in xrn3. However, both the position and the level of Pol II at the peak summit were not affected in xrn3 mutant (Fig. 2c, Fig. S3b, c), which indicated XRN3 doesn’t control the 3’ end Pol II pause to a large extent. Overall, we can’t conclude that pausing is absolutely unaffected by XRN3, but our data showed that such an effect (if any) is quite subtle. In light of the reviewer’s comments, we have checked the result section carefully to make sure our conclusion is fair and accurate.*

I would like the author to discuss the fact that xrn3-3 used by them is a weak allele and xrn3-8 or fry would be more suitable here.

*Response: Thanks for raising this point. We have added discussion about this issue like this: “ It is also worth noting that a relatively weak allele xrn3-3 is used in our study, thus we can’t exclude the possibility that a stronger effect on 3’ pause can be observed in a stronger allele of XRN3 such as xrn3-8. ”*

*In the meantime, it is worth noting that in our pNET-seq data, 11446 genes showed significantly increased readthrough in xrn3-3 compared to Col-0. Thus, although xrn3-3 is a weak allele, this mutant affects termination globally without a significant impact on 3’ end pause.*

Finally the herboxidiene used by the authors to stop the splicing has been used before in plants for the same purpose (Dolata et al 2015). Interestingly authors, reported no effect of herboxidiene on local Pol II elongation at the splice sites with is consistent with the effect of herboxidiene on splicing that feeds on the 3' pausing as reported here.

*Response: Thanks for the valuable suggestion. We have now cited Dolata et al., 2015 and briefly discussed the consistency between the two works in our manuscript (lines 407-411).*

*“Thus, a considerable proportion of on-chromatin splicing could be finished when Pol II pauses at gene 3’ end. Indeed, consistently, it was also reported previously that splicing inhibition did not change the local Pol II dynamics at the splice junction of individual genes (Ref 54). ”*

Reviewer #2: The authors generated Ser2P Pol II positioning data specifically enriched at gene 3' end and defined the 3' end pause index (3'PI) to group genes with or without 3' end pausing. They also incorporated published datasets into their analysis with similar or different treatments. They concluded that the number of exons is a major determinant of Pol II pause and proposed a model to estimate pausing time for genes with different exon numbers.

Major points,
1. The interpretation of pNET-seq need to be improved significantly.

1a, As the major method used in this manuscript, the introduction for pNET-seq (Line 96-100) is hard to follow.

*Response: We apologize for the lack of clarity in writing here. We have improved the introduction for pNET-seq as the following:*

*“In mammals, high precision mapping of Pol II position was enabled by mammalian native elongation transcript sequencing (mNET-seq). In mNET-seq, Pol II is immunoprecipitated together with its associated 3’region of the nascent RNA by using monoclonal antibody that recognize the YSPTSPS repeats of endogenous Pol II CTD with or without phosphorylation at a certain cite. Pol II position in single-nucleotide resolution is then defined by mapping the 3’end of the immunoprecipitated RNA. Notably, due to the conservation of YSPTSPS repeats* *of* *Pol II CTD among eukaryotes[39], the antibody used in mNET-seq could be applicable for a broad range of species. Indeed, by applying mNET-seq in Arabidopsis (named as plant NET-seq, pNET-seq) [40], …”*

*We hope it is now much easier for the readers to follow.*

Line 99, The word "peak" appeared suddenly without stating anything related to immunoprecipitation.

*Response: We have edited the text here as the following:*

*“Indeed, by applying mNET-seq in Arabidopsis (named as plant NET-seq, pNET-seq) [40], it was showed that the Pol II with unphosphorylated CTD (Unphos) is mainly enriched at gene 5’end, albeit such 5’ end Pol II peak is broader than the 5’ end Pol II pausing peak observed in mammals.”*

*We hope the word “peak” does not read odd here.*

Line 101-103, Pol II with Ser5 phosphorylated CTD is related to spliceosome but can not be simply proved by the pNET-seq result using total and Ser5P specific Pol II antibodies.

*Response: We thank the reviewer to point out the issue here, which we totally agree with. In light of the reviewer’s comments, we have edited the text as the following:*

*“Pol II with Ser5 phosphorylated (Ser5P) CTD is closely related with the spliceosome as splicing intermediates that are presumably protected by spliceosome are detected in Ser5P Pol II [39] and Total Pol II NET-seq data (known as plaNET-seq)[40],…”.*

*We hope the edited sentence is fair and accurate now.*

Line 102, Is this "NET-seq" different from pNET-seq?

*Response: Yes, there are differences between pNET-seq and Total Pol II NET-seq. The Total Pol II NET-seq is named as plaNET-seq in plant. In plaNET-seq, FLAG-tagged Pol II is expressed from a transgene and immunoprecipitated with anti-FLAG antibody. To increase the clarity, we have edited the text to make it clear that pNET-seq is somewhat different from plaNET-seq. The edit is shown below.*

*“…splicing intermediates…are detected in Ser5P Pol II [39] and Total Pol II NET-seq data (known as plaNET-seq)[40]”*

Line 105 Please try to re-write "Ser2P Pol II is mainly associated with active elongation and Pol II…"

*Response: Thanks for the comments and our apology for lack of clarity here. We have edited* *the text as the following: “Indeed, evidence from both mammals and plants showed that Ser2P Pol II is mainly associated with active elongation and transcription termination”*

Line 106, Please at least include Pol II CTD with Ser2 phosphorylation.

*Response: Thanks for the comment. We have corrected this issue as suggested by the reviewer.*

*“In Arabidopsis, PTEF-b kinase responsible for Ser2 phosphorylation of Pol II CTD is required for FCA-mediated RNA 3’end processing at FLC gene”.*

Line 107, the upper case FLC usually means a protein.

*Response: We thank the reviewer for the suggestion. Sorry about the possible confusion here. Somehow in plant community, the italic upper case is often used for genes, while the normal upper case is often used for proteins. We hope it is OK to put italic upper case FLC here as a gene symbol.*

Line 107-110, The mammalian genomes are usually 20 times larger than Arabidopsis, and the average transcript length is also 8-10 times. What did the author want to say by comparing "a few thousand base pair " in mammals to "300 bp" in Arabidopsis? "300 bp" too long or too short?

*Response: We appreciate the reviewer for raising this point. Our apology for the possible confusion here. We want to express that Arabidopsis 3’end pausing peak is much narrower than that in mammals. We have modified the text as the following:*

*“Unlike the mammalian Pol II which normally accumulates at a rather broad region over a few thousand base pair post polyadenylation site [43, 47], Arabidopsis Pol II displays a much sharper peak post polyadenylation site often with 250 bp [39, 40, 48], indicating plant Pol II pauses at gene 3’end.”*

*We hope it is much clearer now. Also notably, although the mammalian gene is often 8-10 times longer than the Arabidopsis gene, the Pol II peak in gene 5’ end (presumable 5’end pausing) is significantly broader in Arabidopsis than in mammals. Thus, the relationship between the breath of Pol II peak and gene length is not definitive. Overall, we want to highlight that the transcription termination process in Arabidopsis has its distinct aspect that is different from mammals.*

Line 111, The median size of the termination window detected using nascent RNA sequencing is 161 nt. Why this size coincides with "300 bp"?

*Response: We appreciate the reviewer for raising this point. Sorry about the confusion and we agree with the reviewer that the word “coincides” is not proper here. We have edited the text as the following:*

*“**In addition, the position of Pol II peak at gene 3’end coincide with the end of the termination window as measured by long reads sequencing^1^ (Mo et al, 2021, Genome biology), indicating 3’ end Pol II pausing is likely associated with termination.”*

*We hope this is a fair description of what was reported previously^1^ (the figure 3b from Mo et al. 2021, Genome Biology was shown below).*

Line 114, which Pol II peak? Ser2P/Ser5P or total? Why BORDER is "Consistently" with the rest part of the paragraph?

*We thank the reviewer for the critical reading and constructive comments here. Our apology for the confusion here. The previous paper reported that the 3’ pausing index, as measured by Ser2P, Ser5P and Total Pol II ChIP-seq are somewhat all decreased in the border mutant. By saying “consistently” we wanted to express that the BORDER’s case also showed that 3’ pause is connected with termination in Arabidopsis given BORDER affects both 3’ pause and termination. To increase the clarity, we have edited the text as the following:*

*“Consistent with the above notion, a plant homolog of Negative Elongation Factor called BORDER was reported to promote both 3’end Pol II pause (as detected in Ser2P, Ser5P and Total Pol II ChIP-seq) and termination in Arabidopsis.”*

1b, Line 121, the choices for antibodies for Pol II CTD should be an important part for pNET-seq.
These monoclonal antibodies are designed for mammals. What is the specificity of this antibody (3E10) in plants? Why do the authors prefer 3E10 over CMA602? The selection of antibodies needs to be mentioned in the introduction.

*Response: We appreciate the reviewer’s comment here. Indeed, the choice of the antibody is important for pNET-seq. Both 3E10 and CMA602 antibodies are raised against the repetitive regions in the CTD of Pol II. Given the conservation of YSPTSPS heptapeptide sequence within Pol II CTD between plants and mammals (see below figure a), these antibodies are expected to have very similar specificity against Pol II in both plants and mammals.*

*With the above said, there are considerable differences between 3E10 and CMA602, and there* *are four reasons why we prefer 3E10 over CMA602. First, we noticed that the CMA602 is raised against the* *SYSPTSPSYSphosPTSPSYSP peptide ^2^, which contains two repeats of the heptapeptide and only the second YSPTSPS contains Ser2P. In contrast, 3E10 is raised against YSphosPTSPS heptapeptide. Notably,* *SYSPTSPSYSPTSPSYSP sequence appears less frequently in Arabidopsis Pol II CTD than that in mammals (see below Figure a ^3^). Thus, we expect CMA602 may work less efficiently compared with 3E10 to recognize Arabidopsis Ser2P Pol II CTD. Secondly, according to the technical data provided by the developer of these antibodies (see below Figure b and c). CMA602 has considerable cross-reactivity against the peptide in which both Ser5 and Ser7 are phosphorylated (see below Figure b)^2^. This feature may explain why the CMA602 pNET-seq profile is somewhat similar to the Ser5P profile (enriched at gene 5’end) in Arabidopsis. Thirdly, we have previously tested the 3E10 antibody in Arabidopsis ^4^. This antibody gives an expected pattern of Ser2P Pol II that is enriched at the 3’end of FLC ^4^. A single concrete band of Pol II can be detected by this antibody through immunoprecipitation (see below Figure d or Fig. S1a). In addition, this antibody also showed an exceptional dynamic range such that rough linearity between the Pol II ChIP signal and the Pol II concentration can be observed at FLC ^4^ (see below Figure e). Lastly and perhaps most critically, CMA602 is discontinued by the company, thus it is not available to us as well as other researchers around the world. This prevents us or others to repeat previous results generated by using this antibody.*

*Taking account of the reviewer’s comments, we have briefly mentioned the antibody in the introduction (line 97-105). Due to the comprehensive nature of the issue around CMA602 and 3E10, we have mentioned the reasoning in more detail in the results section (line 132-144, 140-142) and in the discussion (line 355-359). We hope the edits are OK.*

*Figure a. Comparison between Arabidopsis CTD and Mouse CTD. Note that the heptapeptide* *YSPTSPS is conserved in both species.*

*Figure b. The specificity of CMA602 against synthetic SYSPTSPSYSPTSPSYSP peptide as showed previously. Note that CMA602 has significant cross-reactivity against S57 peptide.*

*Figure c. The specificity of 3E10 against synthetic YSPTSPS peptide as provided by the company.*

*Figure d (revised Fig. S1a). Western-blot detection of Ser2P Pol II before and after the immunoprecipitation by using 3E10 antibody.*

*Figure e. Linearity of Pol II ChIP assay from our previous study^4^. (A) Schematic illustration of the experimental procedure testing how the Pol II ChIP signal reflects the change of Pol II concentration locally at FLC. (B) 8WG16 Pol II ChIP signal from different FLC chromatin dilutions at 5′ region of FLC. (C) 3E10 Pol II ChIP signal from different FLC chromatin dilutions at 5′ region of FLC. (B and C) Theoretical values indicate the expected Pol II ChIP signal if the Pol II ChIP signal scales linearly with the local concentration of Pol II. The bar chart on the Right shows the ChIP signal at FLC and control genes from dilution 1. The signals are well above background (at IGN5). Values are mean ± SEM from two independent samples, with data normalized to Actin7 (Left) or %input (Right).*

Moreover, what is the rationale for re-do an experiment using a different antibody other than specificity? Could the difference between the previous publication (ref39) and the current study fall into the confidential interval between replicates? For example, what is the variation between two IP experiments using the same antibody? The author needs to provide replicates to claim the difference.

*Response: We appreciate the reviewer for the comments here. As we stated above, there are several reasons why we prefer 3E10 antibody (see our response to the above point). For the replicates, we further analyzed two biological replicates of our data and previous data ^5^ separately as shown in the figure below. Two replicates of our data (3E10) are highly consistent, while the repeatability is not as good for previously published data using CMA602 antibody. The rep1 from the previous work ^5^ also seems to have insufficient sequencing depth. Albeit* *technical variations, each antibody showed its distinct feature such that only CMA602 pNET-seq profile showed enrichment at gene 5’end in both replicates. Overall, the difference between our data and previous data is unlikely due to technical variation among different IP.*

*Figure. a. Metagene plots indicate two replicates of the pNET-seq profile along the gene, as generated by using 3E10 antibody in our study. b. Metagene plots indicate two replicates of pNET-seq profile along the gene, as generated by using CMA602 antibody in a previous study ^5^ (Zhu et al., Nature Plants, 2018). c. Individual gene examples at replications of pNET-seq by using 3E10 antibody and CMA602 antibody respectively.*

2. The correlation analysis for figure 1 is confusing. What is the cutoff for a significant correlation? The P values are all very small though some of the figures seem to be just random.

*Response: We appreciate the reviewer for raising this point. Sorry for the possible confusion here. Indeed, the p-values are all very small for the analysis in Figures 1e, f, g. For this reason, we found it would be very difficult to set up an arbitrary cutoff for significant correlation. In our analysis, we judged the correlation by using the correlation coefficient (the R) and the p-values. The R indicates how strongly the two factors are associated, while the p-value indicates the significance of such association or correlation. For the case of Figure 1e and g, the R is quite small and the p-value is also small, indicating there is a very weak but technically significant association between 3’ PI and the length of the intragenic region or the termination window size. Take the termination window size as an example, the small p-value indicates termination window size could be a contributing factor of 3’ pause, but due to the very small R, it is at most only a quite minor contributing factor among other contributing factors for 3’ pause. We have consulted with our colleague who is an expert in statistics, and also checked the relevant literature (For example, https://sphweb.bumc.bu.edu/otlt/MPH-Modules/PH717-QuantCore/PH717-Module9-Correlation-Regression/PH717-Module9-Correlation-Regression4.html), we find it is actually not unusual to find a weak but still statistically* *significant correlation, especially when the sample size is quite big like in our case. When p-values are all small, the value of R is a better indicator of a strong or weak/no association. Taking into account the reviewer’s comments, we have modified the main text (see below, updated main text line 171-176), we hope the updated text is much clearer and match the results in Figure 1.*

*“**Consistent with previous observations, the position of 3’ end pause is well-matched with TWS such that the bigger the TWS, the longer distance between 3’ end pause and poly (A) site can be observed (Fig. 1e). Thus, the 3’ end pause is indeed tightly associated with termination. Comparatively, we found that the 3’PI does not or only very weakly correlate with TWS at the genome-wide scale (Fig. 1f), as judged by a correlation coefficient of -0.08.”*

3. The analysis for readthrough transcript and 3' paused transcript is interesting. Can the author calculate and compare the proportion of paused/readthrough from pNET-seq or CB-RNA-seq for better comparison?

*Response: Thank you for raising this point. As the reviewer suggested, we have compared the proportion of paused/readthrough from pNET-seq data. As shown in the updated Figure S3c and 4b (See below). For both xrn3 and fcafpa, the majority of genes that showed increased readthrough compared with Col-0 are paused. This is consistent with our conclusion that 3’end pause is often associated with termination.*

The significance for I would also suggest performing paired comparisons between same genes in Col-0 and fca/fpa mutant instead of using tests for independent variables.

*Response: Thank you very much for the valuable suggestion here. Sorry for not writing our figure legend clearly here. We actually performed the paired Wilcoxon test between the same genes in Col-0 and fca fpa mutant to calculate the significance in Fig.3c, Fig.S4a, and Fig.S4d. We have corrected and changed the figure legend from “two-side Wilcoxon test” to “paired Wilcoxon test” in our manuscript.*

Line 206, The significace between 3'PI index seems mild. And the trend for 3'PI change in all genes and the genes with increased readthrough is the same (Figure 3 and S4), which means the increased readthrough caused by fca fpa mutant has no impact on 3' end pause?
*Response: Thank you for raising this point. We agree with the reviewer that the fcafpa mutant did not affect 3’PI strongly. Indeed, the genome-wide distribution of 3’PI is also slightly reduced in fcafpa. However, comparing fcafpa with Col-0, it is worth noting that genes that show increased readthrough in fcafpa also display a bigger reduction of 3’PI, compared with genes that do not show an altered readthrough in fcafpa (See below figure which is included as updated Fig. S4d). Thus, this data suggest FCA and FPA plays a minor but still significant role in promoting 3’PI. In light of the reviewer’s comments, we have edited the main text (updated line 219-221) such that the conclusion is FCA/FPA only mildly affects 3’PI. This is consistent with our central conclusion, that is, the number of introns or exons is a major determinant of 3’ end pause.*

*Figure. Left: genes with increased readthrough in fcafpa. Right: Genes with unchanged readthrough in fcafpa. 3‘PI are displayed as boxplots in the Y-axis.*

4. The conclusion of 3' end Pol II pause correlates with the number of introns is a tricky part.
The gene length itself still seems to be a better indicator. First, genes has isoforms, the total gene number of all bars in figure 4 c&d is far larger than the gene number (18,038) in figure 1e, indicating that they are using isoforms in figure 3c&d, but not in figure 4e.

*Response: Thank you for raising this point. Sorry about the possible confusion here. We only use one isoform (the longest isoform) for each gene in our analysis. We have added this information into the method section (updated line 588-589). The gene number in Figure 4c and 4e are very similar (18010 for original 4c, 18038 for original 4e). 4e has slightly more genes because there are a few chloroplasts and mitochondria genes being included; we have corrected this issue in the revised version, thus the gene numbers is identical in Figure 4c and 4e (18,010).* *The reviewer is correct that figure 4d has larger numbers of genes compared with 4e, this is because 4d describes the relationship between gene length and intron numbers throughout the genome. Thus, almost all the annotated genes are included in 4d, but many of them might not be expressed or might not be paused to be able to calculate the 3’PI. For this reason, 4d has lots more genes than 4c or 4e.*

Updated Figure 4c, d, e

Second, the correlation analysis in figure S5e also indicates a good correlation in 3'PI and gene length with intron number <=3. And the authors may need to change the correlation analysis in figure S5e from categories to number, which is used in figure 4e.

*Response: Thanks for raising this point. We have updated the Fig. S5e from categories to exon numbers as suggested. The updated analysis (see below figure, now as updated Fig. S6a) also showed that gene length only correlated with 3’ PI when a gene has 0 or only 1 intron. In contrast, when we fixed the gene length, the correlations between 3’ PI and intron number are always good (Fig. 4f). These results indicate 3’ PI is correlated with intron number largely independent of gene length. In other words, although intron number and gene length both correlate well with 3’PI, intron number has more weight for this correlation. As kindly pointed out by the reviewer, gene length by itself might also be a contributing factor to determining 3’PI, especially for intron-less genes. We have edited the text to describe these results more accurately (see below, updated main text line 242-248). We hope the added analysis helped to resolve the relationship between intron number, gene length and 3’PI, such that our conclusion is fair and judged.*

*“In comparison, 3’PI is not correlated with gene length at genes with a fixed number of exons when the exon numbers are above three (Additional file 1: Fig. S6a). Of note, gene length correlates with 3’PI most significantly for single-exon genes. Similar results were obtained when considering the relationship between 3’PI and exon length at gene groups with fixed numbers of exons (Additional file 1: Fig. S6b). Therefore, between gene length and the numbers of exons, the later has a significantly stronger contribution in determining the correlation with 3’PI for multiple exon genes.”*

*Updated Fig.S6a. Scatter plots indicating the relationship between 3’PI and gene length in gene groups with fixed exon numbers. The orange line indicates the trendline fitted based on the least squares method. Spearman correlation coefficient is indicated on top of the plot.*

The splicing defect mutants or the herboxidiene treatment could differentiate the effect of the intron/exon number with gene/isoform length. However, the intron retention also increased the length of the transcript.

*Response: Thank you for raising this point. We use herboxidiene treatment to inhibit splicing, and the result showed that the 3’PI is strongly reduced after the splicing inhibition (Fig. 6b). In addition, the correlation between 3’PI and intron numbers is largely disrupted after the splicing inhibition (Fig. 6c). As the reviewer pointed out, splicing inhibition may make the gene longer in a way. However, if we assume the gene length is the major contributor here, then it predicts that 3’PI should be increased after the herboxidiene treatment. In addition, the correlation between 3’PI and intron number or gene length in association shouldn’t be disrupted. For these reasons, we believe the result of herboxidiene treatment can’t be explained by an increase in gene length.*

5. If the author admit that the gene length rather than intron number is the key to Pol II pausing, the modelling of Pol II occupancy could be simplified by incorporating a decrease of elongation speed alongside the gene. And why the authors have to fit 10 models for genes with different intron numbers?

*Response: We thank the reviewer for the constructive comment.*

*For the first comment, for the reasons we stated above, we believe the intron number instead of the length is the main contributing factor in determining the 3’PI. Hence, in light of the reviewer’s comments, to assess the possibility that the Pol II occupancy profiles we have observed are simply caused by the decrease of elongation speed through the gene body, we have built an alternative model which quantifies the decrease of elongation velocity along the gene using a logistic function:*

$$\boldsymbol{v}\left( \boldsymbol{x} \right)=\frac{\boldsymbol{v}_{\boldsymbol{0}}}{\boldsymbol{1}+\boldsymbol{e}^{\boldsymbol{ax}+\boldsymbol{b}}}$$

*In which* $\boldsymbol{x}$ *is the current location of Pol II and* $\boldsymbol{v}\left( \boldsymbol{x} \right)$ *is the velocity of elongation at position* $\boldsymbol{x}$*.*

*We fit this alternative model to the Pol II occupancy profiles in the Col-0 group, and compared the model-predicted Pol II occupancy profiles to experimental data in genes with altering lengths. We categorized the genes into ten bins based on their gene length, and compare the model predictions to average Pol II occupancy profiles of genes in each bin (see figure below). The fitting results show that the alternative model is not consistent with experimental data, especially for longer genes. Instead of a sharp peak appearing near the PAS, this alternative model predicts that Pol II occupancy slowly increases alongside the gene body, followed by an abrupt drop after the PAS. These results indicate that the assumption that Pol II pausing depends on intron number is important for the model to correctly predict the Pol II dynamics.*

*Response Figure. Comparison between model-predicted and actual Pol II occupancy profiles in genes with different lengths. Simulation was done with the parameter set that fits the experimental data the best (i.e. optimal fit) and parameter sets randomly sampled near the optimal fit.*

*For the second comment, we thank the reviewer for raising that comment and apologize for the confusion here. The reason that we fit ten different models for genes with different intron numbers is because the key assumption of our model is that Pol II dynamics depend on the intron number, thereby genes with different intron numbers should have different kinetic parameters (i.e. F, k, b, and d in Figure 7a of the original manuscript). The ten models have the same mathematical form but different kinetic parameters which were estimated by fitting the model to Pol II occupancy profiles of genes with different intron numbers. We have clarified* *this point in the revised manuscript.*

Minor points:

Line 26, please rewrite "3'PI is a function of gene intron numbers and co-transcriptional splicing efficiency"

*Response: Thank you. We have rewritten this sentence as the following: “3’PI is closely associated with gene exon numbers and co-transcriptional splicing efficiency.”*

Line 93 PacBio *Response: Thank you. We have corrected this.*

Figure 4f, I would suggest putting the count number on top.

*Response: Thank you. We have updated this figure.*

Line 211, Usually we would use the number of exons instead of introns.

*Response: Thank you. We have used the number of exons as suggested throughout the text.*

*Reference*

*1	Mo, W. et al. Landscape of transcription termination in Arabidopsis revealed by single-molecule nascent RNA sequencing. Genome Biol* ***22****, 322, doi:10.1186/s13059-021-02543-4 (2021).*

*2	Stasevich, T. J. et al. Regulation of RNA polymerase II activation by histone acetylation in single living cells. Nature* ***516****, 272-275, doi:10.1038/nature13714 (2014).*

*3	Liu, P., Kenney, J. M., Stiller, J. W. & Greenleaf, A. L. Genetic organization, length conservation, and evolution of RNA polymerase II carboxyl-terminal domain. Mol Biol Evol* ***27****, 2628-2641, doi:10.1093/molbev/msq151 (2010).*

*4	Wu, Z. et al. Quantitative regulation of FLC via coordinated transcriptional initiation and elongation. Proc Natl Acad Sci U S A* ***113****, 218-223, doi:10.1073/pnas.1518369112 (2016).*

*5	Zhu, J., Liu, M., Liu, X. & Dong, Z. RNA polymerase II activity revealed by GRO-seq and pNET-seq in Arabidopsis. Nat Plants* ***4****, 1112-1123, doi:10.1038/s41477-018-0280-0 (2018).*

**Second round of review**

**Reviewer 1**

I think all of the main issues have now been addressed and the manuscript is greatly improved.
I am still a bit confused by the figS13 panel c and d where a change in 3' Pol II pausing can be observed at intron-less genes in fcafpa double as well as the reduction in splice site occupancy in xrn3 (SF13d). I think those should be discussed in the discussion.

It is also a bit confusing how the authors try to incorporate into their model fcafpa and xrn and compare the changes between them. While xrn is a generic factor fcafpa are clearly gene specific. This is acknowledge in the text but could be stressed also in the discussion when the authors interpret the data.

Also the authors show that not all genes have the 3' Pol II pausing. While the assumption is that those are probably the one without introns, it would be nice to look at them specifically and ask how many of them have introns etc.

**Reviewer 2**

Thank the authors for the reply. Some of my questions still need further clarification.

1. The author revised most of the “300bp” to “250bp”, why? It seems to be just an arbitrary length to me now. And most of the analysis still uses ~301 from PAS or ~150+300 from PAS, which is quite an inconsistency.

2.About the question “What did the author want to say by comparing a few thousand base pairs in mammals to 300 bp in Arabidopsis? "300 bp" too long or too short?”.

The authors’ reply deepens my confusion. Their figures used to illustrate a sharper or broader peak is a figure with the same relative length from ATG to Stop codon, which makes the normalization from real length to relative length important. “the Pol II peak in gene 5’ end (presumable 5’end pausing) is significantly broader in Arabidopsis than in mammals” could be just a misinterpretation from the difference of gene length. Because the gene length in manuals

3.The numbers of genes are still not correct. Araport11 has 27655 protein-coding genes. However, the sum in fig 4d has a number of 32399 genes, what is the data source? The number did not match tair10 or Araport11.

**Reviewer 3**

In this manuscript, Zhou et al. presented the genome wide nascent RNA and chromatin bound RNA sequencing data to link co-transcriptional pre-mRNA splicing with 3’ termination. The manuscript is well presented, and the results are mostly solid. However, there is room for improvement in terms of experimentation and analysis, making the conclusions more convincing.
1. Changing the title to “coupling between cotranscriptional-splicing and 3’ pausing in Pol II terminator in Arabidopsis” would probably be straightforward.
2. Line 155-157. It is not clear how the 3’ end peak was defined. What if there are two peaks within the terminator of one gene?
3. I couldn't find information on how to select gene sets, such as Figure 1d-F. Also, some subsequent analyses used different sets of genes.
4. Line 183, based on the results of xrn3-3, it seems that Pol II release from 3’ pause somehow depends on the XRN3? Or XRN3 negatively regulates 3’ pause?
5. Line 198-200. For such a large amount of data, almost half of the gene transcription terminations are disturbed. Then, normalizing the data is critical. I can’t think of a better way to normalize than spike-in RNA.
6. One of the replicates of fca fpa looks funny to me (FigureS13). Especially, only two replicates were performed, it is premature to draw a conclusion based on inconsistent replication. E.g., FigS13 a-c, and f, fca fpa rep1 looks more like the wild type rather than fca fpa rep2.
7. Alternative polyadenylation was interfered by fca fpa mutation. When compare the meta profiles between wild type and fca fpa, the authors appear to pick one transcript isoform for each gene. How can the possible effects of APAs be ruled out and conclusions drawn about the direct effect of FCA FPAs on 3’ pausing?
8. Why did the authors calculate 5’SS and 3’SS separately? For a given intron, 5’SS and 3’SS are supposed to be equal. Does this treatment help to solve the alternative splicing event?
9. Line 284, what about the rest genes?
10. Line 318, Pol II occupancy doesn’t necessarily equal to the transcriptional elongation rate. In one case, many Pol II occupy a gene and elongate at a slow rate. The other is that a handful of Pol II occupy a gene and elongate at a fast rate. Both scenarios could end up with mediocre nascent RNA biogenesis rate. Therefore, when discussing transcriptional elongation rates, both the occupancy of Pol II and the incidence of nascent RNA organisms should be considered, which can be determined by pNET-seq/Pol II ChIP and metabolic labelling/nuclear run assays respectively.

**Authors Response**

**Point-by-point responses to the reviewers’ comments:**

*We thank all three reviewers for their constructive feedback and suggestions. We have revised our manuscript carefully according to the comments and suggestions from reviewers. Below is our point-by-point response to the reviewer’s comments.*

Reviewer #1:

I think all of the main issues have now been addressed and the manuscript is greatly improved.
I am still a bit confused by the figS13 panel c and d where a change in 3' Pol II pausing can be observed at intron-less genes in fcafpa double as well as the reduction in splice site occupancy in xrn3 (SF13d). I think those should be discussed in the discussion.

*Response: Thanks to the reviewer for the comments. For both fcafpa and xrn3, like the reviewer pointed out, the Pol II occupancy in the gene-body region was also affected slightly. For fcafpa, we no longer include this data into the manuscript (we have explained the reason in the response to the point 2 of this reviewer, please also see our response to the point 3 of reviewer 3). For the xrn3, indeed the Pol II level is a bit lower at the exon-intron-exon compared with Col-0. As shown in the figure below, we have plotted the xrn3 and Col-0 data in the same plot for comparison. The reduction of Pol II level is not only at the splice site but also at the exon and intron, suggesting the xrn3 mutation could have changed Pol II dynamics during the elongation. This is also evident from the metagene plot shown in the previous S3a (see below), where Pol II occupancy is slightly lower at gene body in xrn3 compared with Col-0. Given xrn3 mutant do not show visible phenotype under normal growth condition, the mRNA expression level is also largely unchanged genome-wide, which means Pol II initiation should not be altered globally. Thus, a plausible explanation is that Pol II elongation is faster in xrn3 compared with Col-0. Although the exact reason for this remains elusive, the phenomenon indicates that termination and elongation are tightly connected. As suggested by the reviewer, we have added this to the discussion (Lines 343-346).*

It is also a bit confusing how the authors try to incorporate into their model fcafpa and xrn and compare the changes between them. While xrn is a generic factor fcafpa are clearly gene specific. This is acknowledge in the text but could be stressed also in the discussion when the authors interpret the data.

*Response: Thanks for the comments. For each genotype and condition, we divided the genes into different groups according to their gene exon numbers. We then fitted the model to the Pol II data for each group of genes respectively. We agree with the reviewer that fcafpa represents a genespecific factor in 3’end pause regulation. This corresponds to the limited role of fcafpa in regulating pol II termination comparing xrn3 throughout the genome. Considering the comments from reviewers 1 and 3, we decided to remove the part related to fcafpa in the revised version. The larger variation among two repeats of pNET-seq of fcafpa could indeed bring uncertainty when interpreting the data at the genome-wide level. Nevertheless, our data showed fcafpa only had some mild and gene-specific effects on 3’end pausing. Therefore, the data add limited value to the main conclusion of the paper. We therefore would like to thoroughly explore FCA and FPA’s function in transcription termination in future studies.*

Also the authors show that not all genes have the 3' Pol II pausing. While the assumption is that those are probably the one without introns, it would be nice to look at them specifically and ask how many of them have introns etc.

*Response: Thanks for the comments. We have analyzed the numbers of genes without introns in paused and non-paused genes respectively. In 18010 paused genes in Col-0, there are 2269 (12.6%) intron-less genes. In 4232 non-paused genes, there are 1957 (46.2%) intron-less genes. This data is consistent with our main conclusion that intron numbers (co-transcriptional splicing) is one of the main determinants for 3’end Pol II pausing. In light of the reviewer’s comments, we have added this information to the main text (Line 205-207).*

Reviewer #2:

Thank the authors for the reply. Some of my questions still need further clarification.

1. The author revised most of the “300bp” to “250bp”, why? It seems to be just an arbitrary length to me now. And most of the analysis still uses ~301 from PAS or ~150+300 from PAS, which is quite an inconsistency.

*Response: We thank the reviewer for this comment. Our apology for the confusion here, we mentioned 250bp once in the abstract and once in the introduction because such a number wasintroduced in the pNETseq paper published previously. (e.g. In the abstract “… Pol II with a Ser 2P CTD presents a sharp peak within 250 base pairs downstream of the polyadenylation site“ from Zhu et al, 2018, Nature Plants; Please also see our response to point 2 below). We can confirm “250bp” is a citation of what was said in the previous literature but not based on our own analysis. We agree with the reviewer that this number could be somewhat confusing. In order to avoid further confusion, we avoided saying an exact number in the introduction (e.g. In Arabidopsis, RNA Polymerase II (Pol II) often pauses within a few hundred bp downstream of the polyadenylation site… ).*

*In the results section, as the reviewer pointed out, we use -150 to +300bp of PAS as the 3’end region when calculating the 3’ end pause index. As we clarify below, we believe such a parameter is proper to be used in our study. First, as shown in Fig1 in the current manuscript, among the expressed genes, we determined 18010 paused genes, 4232 non-paused genes and 4964 inactive genes. All protein coding genes are included in this case. From the meta-gene plot, there is indeed no Pol II peak at 3’end for the non-paused genes, indicating the identification of paused or non-paused genes is effective. Indeed, the peak summit of the 3’end Pol II peak for the paused gene is around 100bp post-PAS, well within our chosen window size. To further clarify this point, we calculated the position of pausing for each paused gene and looked at their distribution. The analysis showed that the median and the mean value of the pausing position are 93bp and 97.4bp post-PAS, with the Q1 (25%) and Q3 (75%) values of 40bp and 160bp post-PAS respectively. In light of the reviewer’s comments, we have added the pausing position information to Figure S1 and briefly explained in the method section the rationale underlying the definition of the 3’end region.*

2.About the question “What did the author want to say by comparing a few thousand base pairs in mammals to 300 bp in Arabidopsis? "300 bp" too long or too short?”.

The authors’ reply deepens my confusion. Their figures used to illustrate a sharper or broader peak is a figure with the same relative length from ATG to Stop codon, which makes the normalization from real length to relative length important. “the Pol II peak in gene 5’ end (presumable 5’end pausing) is significantly broader in Arabidopsis than in mammals” could be just a misinterpretation from the difference of gene length. Because the gene length in manuals
*Response: We appreciate the reviewer for the comments here. Our apology for the confusion that is still left here. In order to avoid unnecessary confusion, we have edited and shortened the introduction related to this part. We no longer mention such a difference between plants and animals as this is not the focus of our paper. We totally agree with the reviewer that different genome structures could have influenced the Pol II pausing pattern, at least we can not rule out the impact from it. In the previous version, when we talk about the position of Pol II pausing in the introduction, we mostly based on data published by Zhicheng Dong’s group (https://www.nature.com/articles/s41477-018-0280-0/figures/2). As shown below, unphosphorylated Pol II showed a blunt peak within 1000bp post-TSS, while Ser2P Pol II showed a relatively sharp peak shortly post-PAS. In comparison, in the mNET-seq data published by Proudfoot’s lab (Figure 2C, PMID: 25910207), Total Pol II showed a sharp peak likely within 200bp post-TSS. These analyses are plotted against the exact length. Thus, Pol II peak around gene 5’end seems broader in Arabidopsis than in mammals while the Pol II peak at gene 3’end is narrower in Arabidopsis than in mammals. We totally agree with the reviewer that how exactly this is related to different gene structures or lengths between plants and mammals remains unknown.*

3.The numbers of genes are still not correct. Araport11 has 27655 protein-coding genes. However, the sum in fig 4d has a number of 32399 genes, what is the data source? The number did not match tair10 or Araport11.

*Response: We highly appreciate this comment and our apology for the incorrect number here. Nonprotein coding genes including lncRNA, transposable element genes (TE genes) and pseudogenes were also mistakenly included. We have updated the analysis (Updated Figure 3d, see below) such that only 27206 protein-coding genes from 5 chromosomes are included. We used the TAIR10 annotation for our analysis in this case. The updated result does not change any of our conclusions.*

Reviewer #3:

In this manuscript, Zhou et al. presented the genome wide nascent RNA and chromatin bound RNA sequencing data to link co-transcriptional pre-mRNA splicing with 3’ termination. The manuscript is well presented, and the results are mostly solid. However, there is room for improvement in terms of experimentation and analysis, making the conclusions more convincing.
1. Changing the title to “coupling between cotranscriptional-splicing and 3’ pausing in Pol II terminator in Arabidopsis” would probably be straightforward.

*Response: We appreciate all the comments and suggestions from reviewer 3, which are really helpful and constructive. Thanks for this valuable suggestion on the title. We have altered the title as the reviewer suggested to make it more straightforward.*
2. Line 155-157. It is not clear how the 3’ end peak was defined. What if there are two peaks within the terminator of one gene?

*Response: Thank you for this comment. For the definition of the 3’end peak, we first defined the gene body (from the transcription start site (TSS) to -301 of polyadenylation site (PAS)) and 3’ end region (-150 bp to + 300 bp of the PAS). Using 50 bp sliding windows (with a step size of 5 bp), we identified a peak window within the 3’ end region for each expressed gene. We calculate the read counts within each 50bp window and defined the one with the highest value as the 3’ end peak. In case more than one window is given equally and the highest values within the terminator of one gene, we defined the one closest to the PAS as the peak window. We have updated the method section to clearly state how 3’end peak is defined (line 551-574).*
3. I couldn't find information on how to select gene sets, such as Figure 1d-F. Also, some subsequent analyses used different sets of genes.

*Response: We appreciate this comment. Indeed, we agree with the reviewer that the information about the gene sets was not clearly stated in Fig. 1d-f. For Fig.1d, only 3’ paused genes that does not overlap with other genes are used for this analysis. For Fig.1e and f, only genes with known termination window size and also 3’PI are used for the analysis. We have updated the Figure legends (Fig. 1, 3, 4, 5, S1) throughout the manuscript to indicate the gene sets clearly.*

4. Line 183, based on the results of xrn3-3, it seems that Pol II release from 3’ pause somehow depends on the XRN3? Or XRN3 negatively regulates 3’ pause?

*Response: Thanks for this comment. We agree with the reviewer that our data suggest Pol II release from 3’ pause likely depends on the XRN3 at least to some extent. Such a notion is consistent with the latest observation in mammals (the sitting duck model of termination), where Pol II pauses at the 3’end and the exonuclease degrades the transcript and release the Pol II from the template. Instead of a conclusion, we believe this message is more suitable for discussion (line 344-363). This is mostly due to the fact that the 3’ Pause index is only slightly increased in the xrn3 mutant compared with Col-0. It is also worth noting that we used a relatively weak allele of xrn3 (xrn3-3) in our assay given that the null mutant of xrn3 is lethal in Arabidopsis. Thus, our data has limited power in demonstrating the positive role of XRN3 on Pol II release from Pausing. We would like to explore this issue further when an inducible knockout line of xrn3 becomes available. Nevertheless, our data showed clearly that Pol II pausing at gene 3’end is not mediated by XRN3 given 3’PI is even slightly increased in this mutant. We have discussed the implication of the current data in the discussion.*

5. Line 198-200. For such a large amount of data, almost half of the gene transcription terminations are disturbed. Then, normalizing the data is critical. I can’t think of a better way to normalize than spike-in RNA.

*Response: We appreciate the reviewer for the comment here. We agree with the reviewer, that spikein control could be valuable in many cases. In the meantime, for XRN3, as we detail below, we think that the added spike-in should not be essential for data normalization in our case. First, as we mentioned, we have used a relatively weak xrn3 allele (xrn3-3) in our assay. There is very little morphological or developmental defect of this mutant compared with Col-0 (see below Figure). Thus, we do not expect an extensive change in mRNA expression at the global level. Indeed, previous mRNA-seq analysis by others only revealed less than 2000 genes as differentially expressed genes between Col-0 and xrn3-8 (see table below), the latter is a stronger allele compared with xrn3-3. Second, although our pNET-seq analysis revealed more than 10000 genes are somewhat defective in termination, for each individual gene, the amount of Pol II associated with defective termination (e.g. Pol II travels further downstream of 3’pausing peak) only accounts for a small number of all the Pol II at each gene. This is evident from the fact that the Pol II profile looks overall similar between Col-0 and xrn3, and there is only a small increase of Pol II occupancy downstream of the 3’end Pol II pausing peak (Fig S3a as shown below). Combined with the fact that xrn3 mutation does not affect mRNA levels at a global scale (which means at least the firing rate of Pol II should not be altered substantially at a global scale), these data indicate that although there are a large number of genes showed defective termination in xrn3, the total number of Pol II that is actively transcribing within a cell is unlikely to be altered dramatically between Col-0 and xrn3-3. Therefore, we believe further normalization to spike-in should not be essential in this case. Nevertheless, we very much appreciate the reviewer for raising this point, we would like to use the spike-in control when performing pNET-seq for the inducible knockout line of XRN3 in the future, in this case, a more dramatic change of Pol II is expected.*

6. One of the replicates of fca fpa looks funny to me (FigureS13). Especially, only two replicates were performed, it is premature to draw a conclusion based on inconsistent replication. E.g., FigS13 a-c, and f, fca fpa rep1 looks more like the wild type rather than fca fpa rep2.

*Response: Thanks for the comments. We want to clarify that there are two different panels in Fig. S13(see below). Previous S13a showed a metagene plot of pNET-seq data of all the protein-coding genes in Arabidopsis genome, while S13f showed a metagene plot of pNET-seq data of all the genes that display defective readthrough in fcafpa compared with Col-0. As the reviewer pointed out, S13a showed that fcafpa is unlikely to have any global effect on Pol II dynamics as the rep1 is identical to WT. However, in comparison, S13f showed that rep1 and rep2 both display lower levels of Pol II compared to Col-0 at the 3’end peak, albeit the difference is smaller for rep1. We also looked at how the two repeats behave at the individual genes. We found that for the example genes showed in previous Fig. 3d, the altered Pol II profiles can be observed in both repeats of fcafpa. Taken together, we concluded in the previous version that fcafpa has some local while not global effect on pol II 3’end pausing.*

*In the meantime, we do agree with the reviewer that the Rep1 of fcafpa looks more similar to wildtype compared with Rep2 of fcafpa in the metagene plot. In order to avoid unnecessary confusion, we have removed the part related to fcafpa in the revised manuscript. FCA and FPA overall work at only a subset of genes and their effect on 3’end pausing is generally mild. Thus, the central take-home message of this paper won’t be affected by removing this part of the data. We hope to explore the effect of FCA and FPA on termination more thoroughly in the future.*

7. Alternative polyadenylation was interfered by fca fpa mutation. When compare the meta profiles between wild type and fca fpa, the authors appear to pick one transcript isoform for each gene. How can the possible effects of APAs be ruled out and conclusions drawn about the direct effect of FCA FPAs on 3’ pausing?

*Response: Thanks for the reviewer’s comments. As we mentioned above, we have now removed the FCA and FPA from the manuscript. We totally agree with the reviewer that we can’t be sure the effect we see for FCA and FPA are direct on 3’ pausing. For the APA issue, indeed, FCA and FPA were known for their function in regulating the APA at FLC locus as well as on their own-coding genes. However, based on our own data and previously published genome-wide data of fcafpa and fpa mutant, FCA and FPA only played a minor role in APA selection in general. We got some unpublished data including FCA and FPA RNA-binding data genome-wide, which suggest FCA/FPA works more on termination and the APA choice is likely a reflection of the altered termination. We totally agree with the point raised by reviewer that we would like to consider the APA effect about FCA FPA carefully in the future.*

8. Why did the authors calculate 5’SS and 3’SS separately? For a given intron, 5’SS and 3’SS are supposed to be equal. Does this treatment help to solve the alternative splicing event?

*Response: We thank the reviewer for this comment. We agree with the reviewer that 5’SS and 3’SS are supposed to be equal. We calculate both values just to give more confidence to our assay, that is, both 5’SS and 3’SS give similar results. We haven’t tried to use the difference between 5’SS and 3’SS to solve the alternative splicing, this should be durable in detecting altered usage of 5’ splice site or 3’ splice site in theory.*

9. Line 284, what about the rest genes?

*Response: We appreciate the reviewer for the comment here. Line 284 in the previous version refers to the number of genes that were splicing inhibited after herboxidiene treatment. We apologize for the previous version's inaccuracy as the statistical significance filter was not applied correctly. We have double-checked the number here and updated the text and figures accordingly. We monitor the splicing inhibition by performing total RNA-seq (rRNA depleted but without any polyA RNA enrichment). Among 9659 genes of which the expression can be detected in the total RNA-seq dataset (TPM>=1), 8373 genes showed significant splicing inhibition after herboxidiene treatment compared with the control, as judged by gene average intron inclusion levels (Fig. S7c as shown below). The rest 1286 genes remained unaltered for their gene splicing. There is no gene that showed increased splicing efficiency. We also noticed that the gene numbers being detected in this case are a little bit small, we reasoned this is due to the nature of total RNA-seq. It is likely that rRNA depletion is incomplete, limiting the number of genes that can be detected. Nevertheless, the total RNA-seq data was only used to check whether the splicing inhibition had worked, and the data support that this is indeed the case.*

10. Line 318, Pol II occupancy doesn’t necessarily equal to the transcriptional elongation rate. In one case, many Pol II occupy a gene and elongate at a slow rate. The other is that a handful of Pol II occupy a gene and elongate at a fast rate. Both scenarios could end up with mediocre nascent RNA biogenesis rate. Therefore, when discussing transcriptional elongation rates, both the occupancy of Pol II and the incidence of nascent RNA organisms should be considered, which can be determined by pNET-seq/Pol II ChIP and metabolic labelling/nuclear run assays respectively.

*Response: We thank the reviewer for the insightful comment and completely agree that Pol II occupancy and transcriptional elongation rate are two independent variables. Therefore, high levels of Pol II occupancy do not directly guarantee slow elongation rate, or vice versa. This is the rationale behind the mathematical model we developed to quantify the relationship between Pol II occupancy (P(x,t) in Figure 7a) and transcriptional elongation rate (v(x) in Figure 6a). In that model, the elongation rate and Pol II occupancy both depend on the model parameters v0, xPAS, k, and b, which are determined by fitting the model to the pNET-seq data. We have confirmed that all discussions about transcriptional elongation rate are based on the modelpredicted elongation rate instead of the Pol II occupancy profiles.*

*We totally agree with the reviewer that metabolic labeling/nuclear run assays can be used to directly measure the elongation rate in mammalian cells by tracing the running front of Pol II signal at different time points after metabolic labeling. However, this is not durable in Arabidopsis due to very short gene length in general (on average 10 times shorter than mammalian genes). The short gene length prevented the accurate tracing of Pol II running front as Pol II is expected to finish the elongation of a plant gene in only a few minutes. This is the reason why we believe the modeling is very helpful in our case as it gives a prediction/estimation of different parameters of Pol II transcription that is unable to be directly measured in the plant.*

**Third round of review**

**Reviewer 1**

I am convinced my questions are answered now. I would however advice the authors to included some of the gene browser screen shoots in the supplement to show the differences between the xrn3 libraries.
I think the fca/fpa part removal helped the manuscript.
Some small writing corrections to be included shown below:
line 355 "remains challenge " to "remains a challenge "
correct English
"The exact structure and implication of Pol II conformation at gene 3’ end remains challenge to define. Pol II conformation at 3’ end could be much more complex than just the Ser2P of Pol II CTD, although Ser2P is highly enriched at 3’ end."

**Reviewer 3**

In this revised manuscript, the authors have addressed all issues I raised. They have removed the fcafpa part from the manuscript, which eliminate concerns about the reproducibility of both replicates. I agree that the fcafpa data does not provide significant supportive evidence for the stated conclusion.
However, I am still confused with the mathematics simulation of 3’ end pausing as a function of exon number.
1. In the partial differential equation, parameters such as distances from the transcription start site (TSS) and between TSS and polyadenylation site (PS), initiation rate, elongation rate, and termination rate were collectively considered to contribute additively to the occupancy of S2P-Pol II occupancy. However, it should be noted S2P Pol II only accounts for a franction of transcription engaged Pol IIs. For instance, from the GRO-seq data (PMID: 27729530, Fig4a; PMID: 30374093 Fig2a), a preferential accumulation of Pol II was observed in the 3’ end region proximal to the PS as well as downstream of the PS.
2. Is the term 𝐹 ∙ 𝛿(𝑥) indicative of initiation being a function of distance to TSS? If it is the case, is there a specific range for x, say 0-300 bp?
3. For Figure6b, the X-axis coordinates represent the distance from the TSS. It appears that for genes with a certain number of exons, Pol II will pause at a specific distance regardless of whether the Pol II passes through the PS. Should the X-axis coordinates represent the distance from the PS or the genes were selected based on equivalent length?
4. Also, it is recommended to provide more comprehensive details in the Figure 6 legend to facilitating readers’ understanding. Otherwise, the readers will need to refer the method section and understand the parameter of the equation.
Better be consistent with either Pol II or pol II.

**Authors Response**

**Point-by-point responses to the reviewers’ comments:**

Reviewer #1:

I am convinced my questions are answered now. I would however advice the authors to included some of the gene browser screen shoots in the supplement to show the differences between the xrn3 libraries.

*Response: We thank the reviewer for the positive comments on the revision. As suggested by reviewer 1, in the revised manuscript, we have included some gene browser screenshots for Col-0 and xrn3 replicates in Fig. S13.*

I think the fca/fpa part removal helped the manuscript.

Some small writing corrections to be included shown below:

line 355 "remains challenge " to "remains a challenge "

correct English

"The exact structure and implication of Pol II conformation at gene 3’ end remains challenge to define. Pol II conformation at 3’ end could be much more complex than just the Ser2P of Pol II CTD, although Ser2P is highly enriched at 3’ end."

*Response: We have made the corrections as pointed out by reviewer 1.*

Reviewer#2:

In this revised manuscript, the authors have addressed all issues I raised. They have removed the fcafpa part from the manuscript, which eliminate concerns about the reproducibility of both replicates. I agree that the fcafpa data does not provide significant supportive evidence for the stated conclusion.

However, I am still confused with the mathematics simulation of 3’ end pausing as a function of exon number.

*Response: We thank the reviewer for the positive comments on the revision and apologize for the confusion about the mathematical model. In the revised manuscript, we have included more details about the model to address this reviewer’s concerns.*

1. In the partial differential equation, parameters such as distances from the transcription start site (TSS) and between TSS and polyadenylation site (PS), initiation rate, elongation rate, and termination rate were collectively considered to contribute additively to the occupancy of S2P-Pol II occupancy. However, it should be noted S2P Pol II only accounts for a franction of transcription engaged Pol IIs. For instance, from the GRO-seq data (PMID: 27729530, Fig4a; PMID: 30374093 Fig2a), a preferential accumulation of Pol II was observed in the 3’ end region proximal to the PS as well as downstream of the PS.

*Response: We thank the reviewer for pointing out this and completely agree with the reviewer that the S2P-Pol II occupancy profiled by the pNET-seq experiments only quantifies part of the Pol II dynamics. However, regarding the mathematical model about the pause of Pol II, it does not need any assumption about the location where the pause occurs. In the model, the location of pause is treated as a parameter* $x_{PS}$*, which was estimated by fitting the model to the pNET-seq data. Thereby, if Pol II was observed to accumulate upstream of the PAS in the experimental data (e.g. the GRO-seq data in the papers suggested by the reviewer), the optimal parameter* $x_{PS}$ *will also indicate a location upstream of the PAS. The aim of developing the mathematical model is not to accurately determine the location of the pause, but to provide a quantitative perspective to understand the relationship between exon number and Pol II pausing by comparing model parameters such as pausing time between genes with different exon numbers. We have clarified this point in the revised results section (see below).*

***“****The model hypothesizes that the velocity of transcript elongation decreases near the pausing site (PS) that locates close to the PAS, and the extent of the decrease of velocity and the range of genomic region affected by it, as well as the rates of transcription initiation and termination, change with the number of exons included in the genes. Pol II occupancy was then theoretically predicted by solving the steady state of the model. We estimated model parameters by fitting the model to the average Pol II occupancy profiles in genes with different numbers of exons and found that the model is highly consistent with the experimental data (Fig. 6b, Additional file 1: Fig. S9). It is worth noting that the location of the pausing site is only determined by fitting the model to pNET-seq data. Thus, Pol II elongation dynamics that are not reflected in the pNET-seq data, are not accessed or reflected in our simulation (e.g. Pol II peak before PAS as captured in the GRO-seq data [29, 34]). We define a 3’ end pausing time by computing the increase in total time required for completion of transcription in the scenario with 3’ end pause compared to the scenario without 3’ end pause (see Methods) and calculated the pausing time for genes with different exon numbers.****”***

2. Is the term () indicative of initiation being a function of distance to TSS? If it is the case, is there a specific range for x, say 0-300 bp?

*Response: We thank the reviewer and apologize for the confusion about the definition of the mathematical terms here. We believe the reviewer is referring to the term* 𝐹 ∙ 𝛿(𝑥) *here*. The function 𝛿(𝑥) *in that term* 𝐹 ∙ 𝛿(𝑥) *is a delta function, which has the value one if and only if the variable* $x$ *equals to zero. Otherwise, the function* 𝛿(𝑥) *always have the value zero. This term means that the initiation of transcription only happens with the rate constant* $F$ *at the TSS (i.e.* $x=0$*). We have added definition of the delta function* 𝛿(𝑥) *and clarified this point in the revised methods section (see below).*

*“For the genomic region between the transcription start site (TSS) and the pausing site (PS), the dynamics of Pol II is modeled using the PDE below:*

$$\frac{\partial P(x,t)}{\partial t}=F\cdot\delta\left( x \right)-\frac{\partial}{\partial x}(v\left( x \right)P\left( x,t \right))$$

$$\delta\left( x \right)=\left\{ \begin{matrix} 1 & x=0 \\ 0 & x\neq0 \end{matrix} \right.$$

*In which* $P(x,t)$ *is the level of Pol II occupancy at time* $t$ *and genomic position with coordinate* $x$*. For the definition of the genomic coordinate, the site for transcription initiation is defined to have coordinate* $x=0$*, and positions downstream of the TSS have positive genomic coordinates. In this equation, the term* $F\cdot\delta\left( x \right)$ *describes the initiation of transcription which happens with a non-zero rate of initiation F at the TSS and zero rate at other locations. ”*

3. For Figure6b, the X-axis coordinates represent the distance from the TSS. It appears that for genes with a certain number of exons, Pol II will pause at a specific distance regardless of whether the Pol II passes through the PS. Should the X-axis coordinates represent the distance from the PS or the genes were selected based on equivalent length?

*Response: We thank the reviewer for raising this question. In fitting the model, instead of fitting a different model for each specific gene, we chose to fit an “average model” for* *all genes with the same intron number. In fitting this “average model”, we estimated the average length of genes with a certain intron number and used the average gene length as the coordinate of the X-axis. Hence, the fitting results do not mean that Pol II pauses at a specific distance for all genes with that specific exon number. It means that Pol II pauses at the PS, whose average distance to the TSS in that group of genes is shown by the coordinate of the X-axis.*

*The rationale behind that approach includes two points: first, based on the Pol II occupancy data in the previous figures (e.g. Figure 3b, Figure 3c), there is a strong correlation between intron number and the extent of 3’ pause of Pol II. Thereby, different genes with identical intron numbers should have similar dynamics of Pol II binding, movement, pause, and so on, which can be studied by one model. Second, another goal of fitting an average model for all genes with the same intron number is to reduce the noise in the experimental data and to improve the robustness of the resulting model, especially for genes with lower RPKM values in the pNET-seq data. We have included additional clarifications of these in the revised methods section (see below).*

*“In which* $\hat{P}\left( \boldsymbol{p},x_{i} \right)$ *is the Pol II occupancy at coordinate* $x_{i}$ *predicted by the model using parameter set* $\boldsymbol{p}$*,* $P_{obs}\left( x_{i} \right)$ *is the actual Pol II occupancy at coordinate* $x_{i}$ *measured using pNET-seq. For each model, the range for the coordinate* $x$ *was determined based on average length of genes with the corresponding exon number. The experimental data used for the fitting,* $P_{obs}\left( x \right)$*, was generated by computing the average Pol II occupancy over all genes with the same exon number. We used the average occupancy profiles to fit the model to reduce noises in the pNET-seq data and improve the robustness of the model. After the parameter set* $\boldsymbol{p}_{opt}$ *with optimal fitting was found for each model, we first computed the optimal value of the objective function,* $f_{opt}=f(\boldsymbol{p}_{opt})$ *, and then sampled 50,000 random parameter sets in the region* $[0.316\boldsymbol{p}_{opt}, 3.16\boldsymbol{p}_{opt}]$ *using Latin hypercube sampling and kept all parameter sets* $\boldsymbol{p}$ *with* $f\left( \boldsymbol{p} \right)<2f_{opt}$ *for the following calculation of 3’ end pausing time.”*

4. Also, it is recommended to provide more comprehensive details in the Figure 6 legend to facilitating readers’ understanding. Otherwise, the readers will need to refer the method section and understand the parameter of the equation.

*Response: We thank the reviewer for the helpful suggestion. We have included more details in the Figure 6 legend of the revised manuscript (see below).*

***“a*** *Scheme of the mathematical model. The velocity of elongation (*$v$*) is assumed to be a function of the distance to the TSS (*$x$*), and the pause is modelled by a decrease of velocity at the pausing site (PS) near the PAS. Pol II occupancy between TSS and PS and that after the PS is then computed by solving two partial differential equations that link the Pol II occupancy to the velocity of elongation.* ***b*** *Comparison of model-predicted and actual Pol II occupancy profiles in Col-0. Curves indicate the average Pol II occupancy profiles for all genes with a specific exon number estimated from the pNET-seq data, and the shaded areas indicate model-predicted ranges of the Pol II occupancy based on parameters estimated by fitting the experimental data.* ***c*** *Comparison of model-predicted 3’ end pausing time across genes with different numbers of exons in Col-0. The pausing time was computed by the decrease in the total time for the entire process of transcription initiation, elongation, and termination to complete if there was no 3’ end pause.* ***”***

Better be consistent with either Pol II or pol II.

*Response: We have made the corrections as pointed out by reviewer 3.*
